# Clinical Trial of Human Fetal Brain-Derived Neural Stem/Progenitor Cell Transplantation in Patients with Traumatic Cervical Spinal Cord Injury

**DOI:** 10.1155/2015/630932

**Published:** 2015-10-11

**Authors:** Ji Cheol Shin, Keung Nyun Kim, Jeehyun Yoo, Il-Sun Kim, Seokhwan Yun, Hyejin Lee, Kwangsoo Jung, Kyujin Hwang, Miri Kim, Il-Shin Lee, Jeong Eun Shin, Kook In Park

**Affiliations:** ^1^Department of Rehabilitation Medicine, Yonsei University College of Medicine, Seoul 120-752, Republic of Korea; ^2^Research Institute of Rehabilitation Medicine, Yonsei University College of Medicine, Seoul 120-752, Republic of Korea; ^3^Department of Neurosurgery, Yonsei University College of Medicine, Seoul 120-752, Republic of Korea; ^4^Department of Pediatrics, Severance Children's Hospital, Yonsei University College of Medicine, Seoul 120-752, Republic of Korea; ^5^BK21 Plus Project for Medical Science, Yonsei University College of Medicine, Seoul 120-752, Republic of Korea

## Abstract

In a phase I/IIa open-label and nonrandomized controlled clinical trial, we sought to assess the safety and neurological effects of human neural stem/progenitor cells (hNSPCs) transplanted into the injured cord after traumatic cervical spinal cord injury (SCI). Of 19 treated subjects, 17 were sensorimotor complete and 2 were motor complete and sensory incomplete. hNSPCs derived from the fetal telencephalon were grown as neurospheres and transplanted into the cord. In the control group, who did not receive cell implantation but were otherwise closely matched with the transplantation group, 15 patients with traumatic cervical SCI were included. At 1 year after cell transplantation, there was no evidence of cord damage, syrinx or tumor formation, neurological deterioration, and exacerbating neuropathic pain or spasticity. The American Spinal Injury Association Impairment Scale (AIS) grade improved in 5 of 19 transplanted patients, 2 (A → C), 1 (A → B), and 2 (B → D), whereas only one patient in the control group showed improvement (A → B). Improvements included increased motor scores, recovery of motor levels, and responses to electrophysiological studies in the transplantation group. Therefore, the transplantation of hNSPCs into cervical SCI is safe and well-tolerated and is of modest neurological benefit up to 1 year after transplants. This trial is registered with Clinical Research Information Service (CRIS), Registration Number: KCT0000879.

## 1. Introduction

Acute traumatic spinal cord injury (SCI) occurs most commonly in the cervical segments due to the great flexibility of neck. Cervical SCI is a devastating disorder, which can result in quadriplegia, ventilator dependency, a requirement for total assistance with all major functions, and a significant reduction in quality of life. However, there is currently no curative or effective therapy for SCI [1]. Therapeutic transplantation of different types of stem cells and their derivatives, alone or in combination with other treatments, has been reported to improve functional outcome in animal models of SCI, probably through cell replacement, trophic support, facilitation of axonal growth, remyelination, or modulation of inflammation [2, 3]. Based on these experimental findings, although the field of stem cell therapy is in its infancy, some stem cell or cell-based transplantation using bone marrow-derived cells, umbilical cord blood cells, olfactory ensheathing cells, Schwann cells, activated macrophages, or T cells have already been used in patients with SCI [4–12]. To date, the existing data from clinical trials have shown some stem cell or cell transplants to be safe, but with very limited or no therapeutic efficacy. Thus, stem cell therapies are not yet approved for SCI [3, 13].

Neural stem/progenitor cells (NSPCs) are characterized by a capacity for self-renewal, differentiation into multiple neural lineages, and migration toward damaged sites in the central nervous system (CNS), all of which are currently considered to be promising components for SCI repair and regeneration [14–19]. Recent studies have shown that human fetal CNS-derived NSPCs (hNSPCs) implanted into mice with subacute or chronic SCI were found to successfully engraft, migrate, differentiate into oligodendrocytes and neurons, and improve long-term locomotor recovery [20, 21]. This functional recovery was likely to have been mediated through the integration of donor-derived neurons with host neural circuitry and contact with host motor neurons [22, 23]. Based on these preclinical data, a clinical trial by the company StemCells, Inc., is undergoing to assess the effect of the company's proprietary human neural stem cell transplantation into patients with cervical SCI [24].

The objective of this study was to evaluate the safety, tolerability, and neurological status of patients with traumatic sensorimotor complete (AIS grade A, AIS-A) or motor complete (AIS grade B, AIS-B) cervical SCI following transplantation of hNSPCs into the injured cord. The American Spinal Injury Association (ASIA) Impairment Scale (AIS), which forms part of the International Standards for Neurological Classification of SCI (ISNCSCI) examinations, has been widely used for the diagnosis and prognosis of SCI and represents a toolbox of validated outcomes for use in the forthcoming clinical trials [25]. This is a report of the outcome of the trial, 1 year after transplantation. Our results demonstrate that the direct administration of hNSPCs into the injured cervical cord is safe and well-tolerated and of modest benefit neurologically.

## 2. Materials and Methods

### 2.1. Patient Selection

This phase I/IIa open-label and nonrandomized controlled clinical study was reviewed and approved by the Institutional Review Board (IRB) of the Severance Hospital, Yonsei University College of Medicine, Seoul, Korea (Permit number: 4-2005-0057), Korean Food and Drug Administration (Permit number: BM-473), and was monitored by the responsible ethics committees. This study was conducted in accordance with the Declaration of Helsinki (1964). All procedures were performed after obtaining written informed consent. Patients were fully aware of the experimental nature of the treatment, unclear outcomes, and possible side effects, such as pain, spasticity, autonomic dysfunction, worsening of motor or sensory function, infection, tumor formation, and unforeseen adverse events.

Participants were eligible when they were admitted to the hospital if they were between 18 and 60 years old and had AIS-A or AIS-B cervical SCI of traumatic etiology. Patients first underwent spinal cord decompression and stabilization therapy and then rehabilitation. They showed persistent complete or incomplete paralysis below the level of the injury. Exclusion criteria were SCI at multiple levels, spinal vertebral instability, major concurrent medical or neurological illness, substance abuse, psychiatric illness, traumatic brain injury associated with SCI, and concomitant skeletal fracture or joint atrophy. Subjects who had concurrent peripheral nerve, nerve root injury, or accompanying neuropathy that might influence spontaneous recovery and recordings of evoked potentials [26, 27] were also excluded. To verify peripheral nerve injury or neuropathy, peripheral nerve conduction studies (NCS), including median sensory, ulnar sensory, superficial peroneal, sural, median motor, ulnar motor, peroneal, and tibial nerves, were made in all patients with SCI [28]. If there was any abnormality in the NCS, the patient was excluded.

Nineteen patients were selected for hNSPCs transplantation from among those who were admitted to the hospital between May 2005 and August 2008. According to the time window between the injury onset and hNSPCs transplantation, eligible patients were divided into four groups: acute (<1 week), early subacute (1–8 weeks), late subacute (9 weeks–6 months), and chronic (>6 months). In the control group, all 15 patients with traumatic cervical SCI were managed with decompression surgery of the spinal canal and then referred to the rehabilitation clinic of the hospital. They did not receive hNSPCs implantation for SCI. They were randomly selected from AIS-A or AIS-B patients who were admitted to the hospital from May 2005 to April 2008. The inclusion and exclusion criteria for the control group were the same as the transplantation group. Demographic data and clinical characteristics of patients in both groups are presented in Table 1.

### 2.2. Peripheral Nerve Conduction Study

To verify peripheral nerve injury or neuropathy, peripheral nerve conduction studies, including median sensory, ulnar sensory, superficial peroneal, sural, median motor, ulnar motor, peroneal, and tibial nerves, were conducted before transplantation. For median sensory nerve conduction studies, the recording electrode was placed over the second finger and stimulation was done at the wrist between the tendons of the flexor carpi radialis laterally and the palmaris longus muscles medially. For ulnar sensory nerve conduction studies, the recording electrode was placed over the fifth finger and stimulation was done at the wrist, just lateral to the tendon of the flexor carpi ulnaris muscle. For superficial peroneal nerve conduction studies, the recording electrode was placed 1-2 cm medial to the lateral malleolus and stimulation was done at 12 cm proximal to the recording electrode, just anterior to the anterior margin of the fibular. For sural nerve conduction studies, the recording electrode was placed at 3 cm posterior to the lateral malleolus and stimulation was done at 14 cm proximal to the recording electrode, just lateral to the leg midline. For median motor nerve conduction studies, the recording electrode was placed over the abductor pollicis brevis muscle and stimulation was done at the wrist and the antecubital fossa. For ulnar motor nerve conduction studies, the recording electrode was placed over the adductor digiti quinti muscle and stimulation was done at the wrist and just below the medial epicondyle. For peroneal nerve conduction studies, the recording electrode was placed over the extensor digitorum brevis muscle and stimulation was done at the ankle, just lateral to the tibialis anterior tendon and distal to the fibular head. For tibial nerve conduction studies, the recording electrode was placed over the abductor hallucis muscle and stimulation was done at the ankle just posterior to the medial malleolus and the popliteal fossa.

### 2.3. Maintenance and Propagation of Human NSPCs in Culture

Human fetal tissue from a cadaver at 13 weeks of gestation was obtained with full parental consent and approval of the IRB of the hospital (Permit number: 4-2003-0078). In this study, hNSPCs for transplantation were derived from such a single donated fetal brain. The methods of acquisition conformed to NIH and Korean Government guidelines. The freshly dissected telencephalic tissue of a fetal brain was transferred from the Good Tissue Practice (GTP) to the Good Manufacturing Practice (GMP) facility. Brain tissue was in dissociation in trypsin (0.1% for 30 min, Sigma) and seeded into tissue culture-treated 100-mm plates (Corning) at a density of 400,000 cells/mL of serum-free growth medium, which consisted of a 1 : 1 mixture of Dulbecco's modified Eagle's medium and Ham's F12 (DMEM/F12; Gibco), supplemented with penicillin/streptomycin (1% v/v; Gibco) and N2 formulation (1% v/v; Gibco). Mitogenic stimulation was achieved by adding 20 ng/mL of fibroblast growth factor-2 (FGF2; R&D Systems) and 10 ng/mL of leukemia inhibitory factor (LIF; Sigma). Heparin (8 *μ*g/mL; Sigma) was added to stabilize FGF2 activity. All cultures were maintained in a humidified incubator at 37°C and 5% CO_2_ in air, and half of the growth medium was replenished every 3-4 days. Proliferating single cells isolated from the fetal brain gave rise to free-floating neurospheres during the first 2–5 days of growth. Passaging was undertaken every 7-8 days by dissociation of bulk neurospheres with 0.05% trypsin/EDTA (T/E; Gibco). hNSPCs, cultured as neurospheres, were reseeded in fresh growth medium at a density equivalent to ~400,000 cells/mL. Under these culture conditions, hNSPCs continued to proliferate and generate a large number of progenies for over 1 year [29]. For cryopreservation of hNSPCs, trypsinized cells taken at each passage were resuspended in a freezing solution consisting of 10% dimethyl sulfoxide, 50% fetal bovine serum, and 40% growth medium and brought slowly to −140°C. Human neurospheres were continuously expanded from the initial outgrowths to avoid repeated freezing and thawing. Cells were pooled and frozen at each passage (P4-30; passage number 4-30) as primary cell banks and following additional expansion cryopreserved (P7-25) as secondary cell banks.

The growth rate of hNSPCs in the presence of mitogens was assessed* in vitro*. Initially, 4 × 10^6^ cells were plated on tissue culture-treated 100-mm plates. At 5 time points up to 50 days, the cells were harvested with T/E and the average number of hNSPCs was determined by using the trypan blue exclusion method. Cell counts were then plotted versus the time of cells harvest and the doubling time was calculated. The doubling time of hNSPCs was between 4 and 5 days. To analyze the cellular composition of the neurospheres, sectioned neurospheres were immunostained for various neural cell markers. Under proliferative conditions, the majority of cells (~99%) within the neurospheres expressed immature cell markers: nestin, vimentin, glial fibrillary acidic protein (GFAP), Pax6, excitatory amino acid transporter 1 (EAAT1), and Sox2 [29]. These results indicated that the cells within the neurospheres consisted mostly of hNSPCs.

### 2.4. Cell Culture Quality Control

Before releasing hNSPCs for transplantation, in-process quality testing for the cells at each passage was carried out for cellular differentiation pattern, karyotyping, endotoxins, mycoplasmas, bacteria, fungi, or viruses. Endotoxins and mycoplasmas were checked using the Limulus Amebocyte Lysate test and MycoAlert Mycoplasma Detection Assay (Cambrex), respectively. HIV-1/2, HTLV-1/2, hepatitis A/B/C, herpes (HSV-1/2), cytomegalovirus, syphilis, toxoplasmosis, fungi, and aerobic or anaerobic bacterial infection were assessed. At any step, if any sample was detected to be positive, it was discarded immediately. For cytogenetic studies of hNSPCs, a standard G-banding procedure to visualize chromosomes was regularly given by the analysis of about at least 20 metaphases per cultural passage. The data from this study confirmed that hNSPCs were diploid soon after derivation and retained a normal karyotype after long-term passage and cryopreservation. Array-based comparative genomic hybridization (aCGH), a technique that allows for high-resolution genome-wide detection of unbalanced structural and numerical chromosomal alterations [30], was performed regularly using MacArray Karyo (Macrogen, Seoul, Korea) according to the manufacturer's protocol. The data from this study showed no evidence for genomic alteration in hNSPCs.

To evaluate the differentiation potential of hNSPCs into neural cell types, whole neurospheres taken at sequential passages in culture were dissociated into single-cell suspensions and plated directly onto poly-L-lysine-coated, 8-well chamber slides (Nunc) in serum-free medium. After a 7-day period following plating, cells were fixed with 4% paraformaldehyde in PIPES buffer (Sigma) and immunostained with antibodies to Nestin, glial fibrillary acidic protein (GFAP), neuron-specific *β*-tubulin III (TUJ1, polyclonal, 1 : 1000; Covance), NF (pan-neurofilament, 1 : 500; Sternberger), rabbit anti-Olig2 (1 : 500; Millipore), O4 (1 : 30; Chemicon), CNPase (2,3-cyclic nucleotide-3-phosphohydrolase, 1 : 500; Sternberger), choline acetyltransferase (ChAT; 1 : 200; Chemicon), glutamate (1 : 500; Sigma), *γ*-aminobutyric acid (GABA, 1 : 500; Sigma), and tyrosine hydroxylase (TH, 1 : 50; Chemicon). Primary antibodies were labeled with a fluorescent secondary antibody. Cells were examined and quantified microscopically and photographed digitally.

About 40–50% of cells from the neurospheres at P10-20 expressed early neuronal cell marker TUJ1, ~2% of cells expressed early oligodendrocyte marker O4, and more than 80% of cells expressed astrocyte marker GFAP. Although the percentages of GFAP^+^ cells from hNSPCs were very high, more than 90% of the cells were dual-labeled with human nestin immature cell marker. Thus, the high percentages of GFAP^+^ cells in hNSPCs do not reflect their differentiation into astrocytes, but they suggest that cells still retain many characteristics of stem cells or progenitors. Before transplantation, the differentiation patterns of hNSPCs taken at between P10-20 were examined immunocytochemically to identify the multipotency of differentiation. In this study, all transplanted hNSPCs met these criteria of differentiation.

### 2.5. Preparation and Transplantation of Human NSPCs

hNSPCs were maintained by passaging through the dissociation of bulk neurospheres and cryopreserved at each passage in the GMP facility. For transplantation, hNSPCs taken between P10 and P20 were selected and prepared. On the day of transplantation, cells were harvested by trypsinization after which the enzymatic activity was stopped with soybean trypsin inhibitor (Sigma). The cells were centrifuged (900 g, 3 min), the cell pellet was washed three times with Hank's balanced salt solution-HEPES (H-H) buffer, and the entire cell pellet was then resuspended in 1.0 mL of H-H buffer at a density of 1.0 × 10^5^ cells per *μ*L. The concentrated hNSPCs in a sterile freezing tube (Nunc) were then delivered to the operation room [31].

Surgical intervention was performed under general anesthesia with endotracheal intubation. All surgical procedures were performed by the same neurosurgical team. A midline incision and posterior laminectomy was performed to expose the dura at the site of injury. Using magnification of an operating microscope (Zeiss Corporation), a midline durotomy was performed away from the site of injury and the opening completed by splitting the normal dura and sharp dissection. A dorsal adhesiolysis was carefully performed using sharp and blunt dissection methods through the injury to remove the scar tissue and detether the cord. After exposure of sufficient surface at the contusion site, cells (1.0 mL, 1.0 × 10^5^ cells per *μ*L) were injected into the spinal cord using a 23-gauge needle attached to a 1-mL syringe. Five hundred microliters of cell suspension was injected at the lesion center, as demonstrated on preoperative magnetic resonance imaging (MRI), and then 250 *μ*L was injected, 5 mm rostral and caudal to the lesion center, respectively. The needle was set at the lesion center along the midline and inserted into the cord 5 mm deep from the dorsal surface of the spinal cord. The needle was removed from the first injection site and moved on the midline 5 mm rostrally and caudally and inserted into the cord 5 mm deep from the dorsal surface of the spinal cord. Each injection was performed over a 3-min infusion period. To prevent cell leakage through the injection track, the injection needle was left in position for additional 2 min after completing the injection. The dura was primarily closed with absorbable sutures and a wound drain was placed. The wound was then closed in layers. Sham operations in the control group were not considered because of the difficulty of ethical justification, given that this would entail an increased risk for the placebo group.

Although NSPCs are minimally immunogenic, low-grade rejection of transplanted NSPCs remains possible [32]. Therefore, many investigators believe that some form of temporary immunosuppression is necessary to optimize fetal donor cell engraftment and survival in humans [33]. For the induction of temporary immunosuppression in this study, cyclosporine (3 mg/kg BID, Novartis, Korea) was orally given to patients for 3 days preoperatively, and it was intravenously given for 4 days after the transplant and orally supplied for the next 2 weeks. The oral cyclosporine dose was reduced to 2 mg/kg BID at 4 weeks after implantation and then reduced to 1 mg/kg BID 3 weeks later and discontinued at 9 weeks postoperatively. Toxicity monitoring included checks of cyclosporine blood trough concentrations, urine analysis, blood urea nitrogen, and creatinine every 48 h during the first week after surgery, biweekly until 5 weeks, and then at the cessation of drug administration. No patient showed side effects due to the cyclosporine.

### 2.6. Outcome Measures

Preoperative and postoperative assessments included AIS neurological examination, according to the revised 2006 ISNCSCI assessments guidelines [25], somatosensory evoked potentials (SSEPs), motor evoked potentials (MEPs), spinal cord magnetic resonance imaging (MRI) scanning, and pain and spasticity assessments. All assessments were made by physicians specially trained for AIS who also had expertise in treating SCI patients. Variability was reduced by using the same assessors throughout the study, obviating interobserver variability.

### 2.7. Neurological Assessments

The neurological status of the patients was determined in terms of AIS grade, ASIA motor scores (AMS), ASIA sensory scores (ASS), ASS-pin prick scores (ASS-P), ASS-light touch scores (ASS-L), and neurological level prior to and at 2, 6, and 12 months after hNSPC transplantation. A five-scale subdivision of the AIS grade was used to evaluate changes in patient motor and sensory function. AIS-A grade has no motor or sensory function at the level of S4-S5 sacral segments. AIS-B has some sensory function below the neurological level, including S4-S5, but no motor function. AIS-C has some motor function below the neurological level, but more than half of the key muscles involved have a muscle strength score that is less than 3. AIS-D has motor function below the neurological level, but more than half of the key muscles have a muscle grade of 3 or more. AIS-E indicates normal motor and sensory function. AMS involves a qualitative grading of the strength of contraction within 10 representative key muscles, five for the upper extremity (upper extremity motor score (UEMS)) and five for the lower extremity (lower extremity motor score (LEMS)) on each side of the body. ASS involves a qualitative grading of sensory responses to touch (ASS-L) and pin prick (ASS-P) at each of 28 dermatomes along each side of the body [25].

The neurological level of spinal injury is generally the lowest segment of the spinal cord with normal sensory and motor function on both sides of the body. Motor level is defined as the most caudal spinal level, as indexed by the key muscle group for that level having a muscle strength of 3/5 or above while the key muscle for the spinal segment, immediately above, is normal (5/5; right/left side). In this study, all AIS-A patients and a motor level of C4–C7 at the baseline assessment in both transplantation and control groups were included in the analysis of motor level. C4 motor level of SCI was determined based on the normal preservation of sensory function on the C4 dermatome [34]. Because there is no key muscle delineating the C4 spinal segment, it is difficult to reliably track deterioration from an initial C4 motor level. Thus, these individuals were analyzed separately for changes in motor level. However, individuals with an initial C4 motor level SCI were included in the analysis of motor level from baseline. An analysis of motor level (right and left side) changes was also performed for the combined group of C5–C7 SCI patients. Sensory level is defined as the spinal segment corresponding with the most caudal dermatome having a normal score for both pin prick and light touch.

### 2.8. Electrophysiological Studies

To assess the functional integrity of the corticospinal tract and the dorsal columns, SSEP and MEP studies of the lower and upper limbs were conducted prior to and at 2, 6, and 12 months after transplantation.

For SSEP studies of median, ulnar, tibial, and peroneal nerves, stimulating electrodes were placed over the median nerve at the wrist, ulnar nerve at the wrist, tibial nerve at the medial ankle, and peroneal nerve at the popliteal fossa, respectively. For pudendal nerve SSEP studies, the stimulating electrode was placed on the shaft of the penis by a ring electrode in males or on the clitoris by a bar electrode in females. Recording electrodes were placed on the C3′-Fz for median and ulnar nerve SSEP studies. For tibial, peroneal, and pudendal nerve SSEP studies, recording electrodes were placed on the Cz′-Fz. The stimulation frequency was 3 Hz and the stimulation duration was 0.1 ms. The stimulation intensity was able to produce a visual contraction of the abductor pollicis brevis (APB) for the median nerve, the abductor digiti quinti (ADQ) for the ulnar nerve, the abductor hallucis (AH) for the tibial nerve, and the extensor digitorum brevis (EDB) for the peroneal nerve. The sweep speed was 5 ms/division and sensitivity was 2 *μ*V/division. With median nerve and ulnar nerve SSEPs, we obtained N20 latency by applying 250 repeated stimulations twice each. For the tibial, peroneal, and pudendal nerve SSEPs, P40 latency was acquired through 250 repeated stimulations that were applied twice. SSEP was performed using Synergy (Medelec Synergy-Oxford Instruments, UK). Normal values are median SSEP: 16.9–20.6 ms, ulnar SSEP: 18.1–20.5 ms, tibial SSEP: 32–46 ms, peroneal SSEP: 32.3–36.3 ms, and pudendal nerve SSEP: 40.4–44.2 ms for men and 38.5–41.1 ms for women. A positive SSEP response was defined as the presence of a cortical response (prolonged latency time or normalization of SSEP) to peripheral stimulation at 1 year after hNPSC implantation, while there was no response before transplantation.

For MEP studies, transcranial magnetic stimulation was performed using Magstim (Magstim Company Limited, UK). The surface recording electrodes were placed over the biceps brachii and abductor pollicis brevis (APB) muscles for the upper limbs and over the tibial anterior (TA) muscle for the lower limbs [28]. The resting motor threshold was the lowest transcranial magnetic stimulation (TMS) intensity that could yield MEP more than 50 *μ*V in amplitude in muscles at rest in at least 5 of 10 stimulations; it was established by increasing the stimulus intensity slowly. We then stimulated the motor cortex 10 times at 1.2 times the intensity of the resting motor threshold and obtained a mean amplitude and latency of MEP. Normal values are biceps brachii MEP: latency 9.1–14.7 ms, amplitude 0.21–1.08 mV; APB MEP: latency 12.2–18.4 ms, amplitude 0.25–1.10 mV; TA MEP: latency 20.2–32.5 ms, amplitude 0.19–0.88 mV. A positive MEP response was defined as the presence of a peripheral response (prolonged or normal latency time, or low or normal amplitude) to transcranial stimulation at 1 year after NSPC implantation, while there was no response before transplantation.

### 2.9. Spine MRI Studies

Spine MRI studies were conducted prior to and at 2, 6, and 12 months after transplantation. MRI examinations were performed with a 1.5/3.0-T magnet using T1- and T2-weighted images (WI) (Signa, GE Medical Systems). For the classification of MRI patterns of acute SCI before hNSPC transplantation, criteria based on alterations in the signal intensity in the spine MRI, as detected by T1- and T2-WI (weighted images) sequences, with respect to time elapsed since the trauma, were used [35]. These criteria are as follows: Type I pattern (hemorrhage): within the first 72 h after the trauma, the spinal cord on T1-WI is heterogeneous; on T2-WI, there is a large central area with low signal intensity surrounded by a thin high intensity peripheral ring. At 72 h to the first week from the trauma, the spinal cord shows hyperintensity on T1- and T2-WI. Type II pattern (edema): there are normal images on T1-WI with high signal intensity on T2-WI. Type III pattern (contusion or mixed): there are normal images on T1-WI, while, on T2-WI, the spinal cord presents with a small central area of isointensity and a thick peripheral ring of high intensity, which persists through the subacute phase. Type IV pattern (compression): there is severe obliteration of the spinal cord with significant alteration of its morphology.

To assess posttraumatic abnormalities and possible complications arising during chronic SCI, MRI findings at 1 year after implantation were evaluated and classified as follows: cord atrophy, myelomalacia, cyst, or syrinx [36]. Cord atrophy is abnormal narrowing of the cervical cord region in the sagittal plane two segments or more beyond the vertebral injury (<7 mm in anteroposterior dimension). Myelomalacia is an area of ill-defined contours and irregular shapes, which is hypointense on T1-WI and hyperintense on T2-WI. Cyst is an oval or round intramedullary lesion with the same signal intensity as cerebrospinal fluid, which is confined to the vertebral level of maximum bony protrusion into the spinal canal. Syrinx is a tubular and well-defined fluid-filled region, which is usually tapered at one or both ends and extends beyond the length of maximal bony damage.

The location of SCI was named for the nearest vertebral segment [37]. Each segment was subdivided into three parts: the upper half of the vertebral body was named segment 1 (e.g., C4.1), the lower part of the vertebral body segment is 2 (e.g., C4.2), and the intervertebral disc below the body segment is 3 (C4.3). In all patients, the lesion length was determined where intramedullary cord signal intensity change was depicted on T1- and T2-WI. Lesion length was defined as the distance between the most cephalic and the most caudal extent of the cord signal intensity change.

### 2.10. Pain and Spasticity Assessments

The development of pain and spasticity in patients following hNSPC implantation was evaluated serially. Pain was assessed using a visual analog scale (VAS) [38]. The VAS frame used a 10-cm bar. Patients indicated their pain score from 0 to 10; zero means no pain and ten means the worst pain imaginable.

Spasticity was evaluated clinically using a modified Ashworth scale with definitions as follows [39]: 0 = no increase in tone, 1 = slight increase in muscle tone, manifested by a catch and release or minimal resistance at the end of the ROM when the affected part(s) is moved in flexion or extension, 1+ = slight increase in muscle tone, manifested by a catch, followed by minimal resistance throughout the remainder (less than half) of the ROM, 2 = more marked increase in muscle tone through most of the ROM, but the affected part(s) easily moved, 3 = considerable increase in muscle tone, passive movement difficult, and 4 = affected part(s) rigid in flexion or extension. Patients were allowed to take medications for the control of spasticity or pain according to their needs.

### 2.11. Western Blot and PCR

hNSPCs were lysed in tissue protein extraction reagent (Thermo) containing protease and phosphatase inhibitors (Sigma), and lysates were centrifuged (16,000 ×g, 30 min, 4°C). The supernatant was collected and stored at −70°C. Protein concentrations were determined using the Bradford assay. Samples were electrophoresed in 10% Tris-glycine gels, 4−15% Mini-PROTEAN TGX precast gels (Bio-Rad), or 16.5% Tris-tricine gels and transferred to nitrocellulose membranes. After being blocked with 5% skim milk or BSA in TBS containing 0.1 or 0.05% Tween 20 (TBS-T), the membranes were incubated with the following antibodies: rabbit anti-neurotrophin-3 (NTF3; Santa Cruz Biotechnology), rabbit anti-nerve growth factor (NGF), rabbit anti-brain-derived neurotrophic factor (BDNF; Santa Cruz Biotechnology), rabbit anti-neurotrophin-4 (NTF4; Santa Cruz Biotechnology), and mouse anti-human vascular endothelial growth factor (VEGF; BD). Next, the membranes were incubated with peroxidase-conjugated antibodies (Jackson ImmunoResearch) and treated with SuperSignal West Pico or Dura chemiluminescent substrate (Thermo).

For PCR analysis, total RNA was isolated from hNSPCs under proliferation and differentiation conditions* in vitro* using TRI reagent (Molecular Research Center). The RNA quantity was spectrophotometrically determined, and 4-*μ*g isolated RNA was reverse-transcribed into cDNA using SuperScript III Reverse Transcriptase (Invitrogen). Reverse transcriptase PCR was carried out in a 20-*μ*L reaction mixture containing 1-*μ*L cDNA following cycle parameters: 30 s at 95°C, 30 s at 53°C for 30 s, and 30 s at 72°C for 31 cycles. Forward and reverse primers were designed to evaluate the expression levels of trophic factors in hNSPCs (*GAPDH*: sense, 5′ ACCACAGTCCATGCCATCAC 3′; antisense, 5′ TCCACCACCCTGTTGCTGTA 3′;* BDNF*: sense, 5′ AACAATAAGGACGCAGACTT 3′; antisense, 5′ TGCAGTCTTTTTGTCTGCCG 3′;* NTF3*: sense, 5′ TACGCGGAGCATAAGAGTCAC 3′; antisense, 5′ GGCACACACACAGGACGTGTC 3′;* NTF4*: sense, 5′ CCTCCCCATCCTCCTCCTTTT 3′; antisense, 5′ ACTCGCTGGTGCAGTTTCGCT 3′;* VEGFA*: sense, 5′ CCATGGCAGAAGGAGGAGG 3′; antisense, 5′ ATTGGATGGCAGTAGCTGCG 3′;* GDNF*: sense, 5′ CTGACTTGGGTCTGGGCTATG 3′; antisense, 5′ TTGTCACTCACCAGCCTTCTATT;* FGF2*: sense, 5′ GTGTGCTAACCGTACCTGGC 3′; antisense, 5′ CTGGTGATTTCCTTGACCGG 3′;* NGF*: sense, 5′ ATGTCCATGTTGTTCTACACT 3′; antisense, 5′ AAGTCCAGATCCTGAGTGTCT 3′). qPCR was performed in 384-well plates using 0.5-*μ*L cDNA in a 10-*μ*L reaction volume with LightCycler 480 SYBR Green I Master mix (Roche) on a LightCycler 480 System (Roche) as follows: 95°C for 5 min and 45 cycles of 95°C for 10 s, 60°C for 20 s, and 72°C for 15 s, followed by a melting curve program. The forward and reverse primers were designed following the PrimerBank database and RTPrimerDB [40].

### 2.12. Preclinical Studies

Thoracic spinal cord contusion injuries were performed on adult male Sprague-Dawley rats. Rats were housed in groups of 4-5 under a 12-h light/12-h dark cycle at 22°C, fed* ad libitum*, and maintained in a facility accredited by the Association for the Assessment and Accreditation of Laboratory Animal Care International. This study was performed under a protocol approved by the Institutional Animal Care and Use Committee, Yonsei University College of Medicine, Seoul, Korea. Spinal cord contusion was performed under Ketamine (80~90 mg/kg), Rompun (0.2 mL/kg), and Promazine (0.8~1 mg/kg) anesthesia and prophylactic administration of cefazolin (50 mg/kg). A dorsal laminectomy was performed on the T9 to expose the spinal cord. Contusion injury was induced using the Infinite Horizon Impactor (Precision Systems, Kentucky, IL, USA) with a force of 230 Kdyn. After contusion, the deep and superficial muscle layers were sutured. Animals received manual bladder expression twice daily and were inspected for weight loss, dehydration, and distress with appropriate veterinary care as needed.

Both cell- and vehicle-injected groups received intraperitoneal injections of 10 mg/kg/d cyclosporine a day before injection and then daily for the duration of the study. Cell- or vehicle-injection occurred 7 days after the induction of SCI. Animals were anesthetized as above and the laminectomy site was reexposed. Totally, 12 *μ*L of hNSPCs (8 × 10^4^ cells/*μ*L) suspended in H-H buffer was slowly injected along the midline of the spinal cord at a depth of 1.2 mm into one segment cranial and one segment caudal to the lesion epicenter using a 26-gauge needle. Control animals received an equal volume of H-H buffer at the same injection rate.

Animals were sacrificed, perfusion fixed, and their spinal cord removed on 12 weeks after transplantation. Fixation was accomplished using 4% paraformaldehyde (PFA; Sigma) in 0.1 M PIPES buffer, pH 6.9, within 24 hours after fixation followed by immersion and sinking in 30% sucrose in PBS. Spinal cords were cut into 16 *μ*m coronal section. The sections were blocked with 3% BSA and 10% normal horse serum with 0.2% Triton X-100 and incubated with primary antibodies to mouse *α*-human nuclei (1 : 100; Chemicon), mouse *α*-nestin, rabbit *α*-GFAP, rabbit *α*-*β*-tubulin III, rabbit *α*-olig2 (1 : 500; Millipore, Billerica, MA, USA), and rabbit *α*-APCCC1 (1 : 50; Abcam, Cambridge, MA, USA). Following the rinsing in PBS, the cultures were incubated with species-specific secondary antibodies conjugated with fluorescein (1 : 180; Vector) or Texas Red (1 : 180; Vector) secondary antibodies.

For the evaluation of hind limb motor function, Basso-Beattie-Bresnahan (BBB) locomotor rate scaling was performed prior to cell- or vehicle-injection and weekly following injection until sacrificed. For the evaluation of nociceptive ability of animals, the Von Frey test was performed. The behavioral responses were used to calculate the 50% paw withdrawal threshold by increasing and decreasing stimulus intensity between 0.4 and 26 g equivalents of force and estimated using a Dixon nonparametric test.

### 2.13. Statistical Analyses

Statistical analyses included the Mann-Whitney *U* test for nonparametric variables between the transplantation and control groups. Fisher's exact test was used to analyze nominal or ordinal variables. Absolute differences between baseline and end values were calculated for AMS, UEMS, LEMS, ASS-P, and ASS-L and analyzed using the Wilcoxon signed rank test for both transplantation and control groups. In the transplantation group, VAS scores were also measured prior to and at 2, 6, and 12 months after implantation to assess pain. These data were analyzed using a one-way repeated measure analysis of variance (ANOVA). All tests were considered significant at *p* values < 0.05. Statistical comparisons were made using the SPSS software (ver. 18.0; SPSS, Chicago, IL, USA).

## 3. Results

### 3.1. Patients

Nineteen patients were enrolled for hNSPC transplantation and followed over 1 year after implantation (16 men, 3 women). All had SCI between C3 and C8 of traumatic etiology. Seventeen patients were AIS-A and two were AIS-B before transplantation. The mean age of the patients was 37.2 (range: 18–57) years and hNSPC transplantation was performed from 16 to 213 days after SCI (mean: 63.4 days). In the control group, 15 patients were included and followed neurologically for 1 year after the initial evaluation of AIS neurological examination in the rehabilitation clinic of our hospital (12 men, 3 women). All had SCI between C3 and C7 of traumatic etiology. Thirteen patients were AIS-A and two were AIS-B. The mean age of the patients was 37.3 (range: 22–56) years, and the time between the injury onset and the initial evaluation of AIS neurological examination was 55.9 days on average (range: 7–168 days). The patients are listed according to AIS grade and baseline characteristics of the patients are summarized in Table 1. Age, gender, and duration from the injury onset to transplantation and to the initial evaluation of neurological examination, neurological level of injury, and AIS grade did not differ significantly between the transplantation and control groups (*p* > 0.05 for each).

### 3.2. Safety Issues

There was no mortality. No patient experienced infection, leakage of cerebrospinal fluid, serious life-threatening autonomic dysreflexia, or progressive spinal deformity following hNSPC implantation. There was no deterioration in sensory and motor function, urinary bladder complications, or neurological level postoperatively due to direct cell injection into the injured spinal cord. No patients showed worsening of respiratory function and all participants appeared to be coping well.

### 3.3. Pain

**Figure 1:**
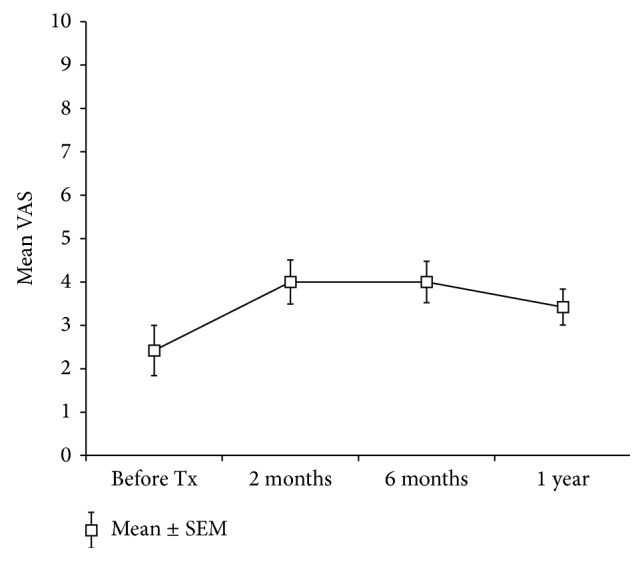
Visual analog scores (VAS).

It has been suggested that pain is a frequent and major consequence of SCI. Estimates of study participants experiencing chronic, disabling pain that interfered with daily activity ranged from 39% to 84% [41, 42]. Furthermore, some studies have reported that cell transplantation strategies increased the risk of neuropathic pain postoperatively [14, 43]. To identify the potential risk of neuropathic pain under the current protocol, pain was assessed prior to and at 2, 6, and 12 months after transplantation with a 10-cm VAS, ranging from 0 to 10. The mean baseline VAS for the 19 patients was 2.4 ± 0.6 (mean ± standard error of mean (SEM)), and, following implantation, the mean VAS changed to 4.0 ± 0.5, 4.0 ± 0.5, and 3.4 ± 0.4 at 2, 6, and 12 months, respectively (Figure 1). No statistically significant difference was found between the baseline and follow-up times (*F* = 2.918, *p* = 0.066), although hNSPC implantation tended to increase mean VAS values at 2- and 6-month follow-up times compared to those prior to cell injection. These results indicate that hNSPCs transplantation was not associated with a greater risk of developing neuropathic pain in patients with SCI, compared with the general population of SCI patients.

### 3.4. Spasticity

Spasticity, defined as increased muscle tone with hyperexcitability of flexor and extensor muscles, exaggerated reflexes, weakness, and joint contractures, is a common complication of SCI [25]. Spasticity was self-reported by 59% and 71% of study participants with SCI [42, 44]. The principal clinical outcome measure for spasticity has been the long-established Ashworth Scale or the modified Ashworth scale, although both scales have less-than-ideal interrater reliability [39] and have a poor correlation with self-rated assessments of spasticity [45]. In this study, to recognize the potential risk of spasticity associated with hNSPC transplantation, spasticity was measured using the modified Ashworth scale in the upper and lower extremities of the 19 patients prior to and at 2, 6, and 12 months after transplantation (Tables 2 and 3). Collectively, only one patient (patient 14) appeared to have clinically significant spasticity in both upper and lower extremities, which affected the activities of daily living. However, he already showed a marked increase in muscle tone of the lower extremities prior to cell implantation and his spasticity was not well controlled with medications over 1 year. Other patients who developed spasticity or displayed an increase in muscle tone during follow-up times did not demonstrate serious spasticity. Their spasticity was relieved with medications and did not have significant effects on their activities of daily living. Thus, given the reported incidence of spasticity associated with the SCI condition, as described above, these results suggest that hNSPC transplantation is not associated with a greater risk of developing spasticity in patients with SCI, compared with the general population of SCI patients.

### 3.5. ASIA Assessments

**Figure 2:**
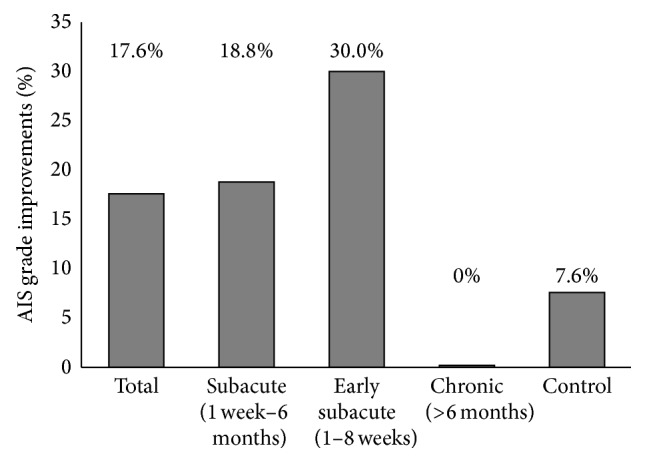
Percentage of individuals converting from sensorimotor complete (AIS-A) to incomplete cervical SCI (AIS-B or AIS-C) in the transplantation and control groups.

The ASIA Impairment Scale has become a standardized and routinely adopted classification for most patients suspected of suffering a SCI [46]. Data obtained using AIS, AMS, and ASS are summarized in Tables 4 [Table tab5] [Table tab6] [Table tab7] [Table tab8]–9.  Figure 2 provides histograms of the estimated mean change in the percentage of patients converting from complete SCI to incomplete SCI. Overall, three (17.6%) of the 17 AIS-A patients improved their AIS grades at 1 year after transplantation: two patients (patients 7 and 15) improved to AIS-C and one (patient 8) improved to AIS-B. If AIS-A patients are classified according to the time window between the injury onset and hNSPC transplantation, 18.8% (3/16) of the patients in the subacute treatment group and 30.0% (3/10) of the patients in the early subacute treatment group showed AIS grade conversion. However, both AIS-B patients (patients 18 and 19) improved to AIS-D at 1 year after transplantation. In contrast, in the control group, only one of the 13 AIS-A patients (patient 6) showed AIS grade conversion (AIS-B) at 1 year (Table 4).

**Figure 3:**
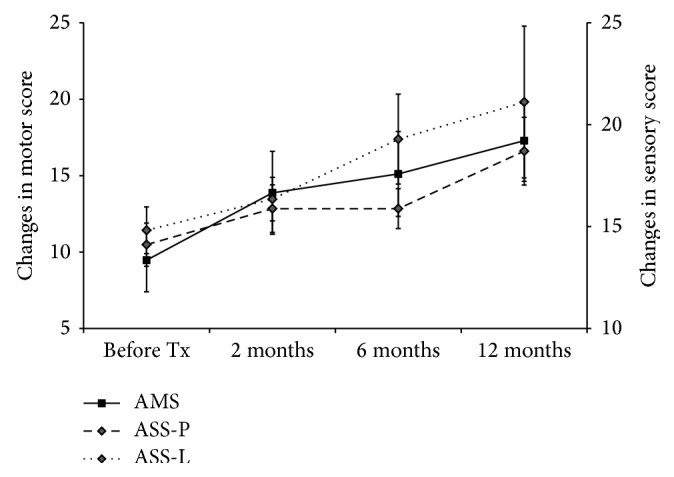
Changes in motor and sensory score over time in patients with complete cervical SCI following hNSPC transplantation. The cervical cohort of patients was followed for 1 year. The mean (mean ± SEM) change in AMS, ASS-P, and ASS-L is shown at each time point (before transplantation (before Tx) and 2, 6, and 12 months after transplantation).

The changes in data obtained using ASIA scores between the baseline and the 1-year follow-up in the transplantation and control groups are summarized in Tables 6–9 and Figure 3. In AIS-A patients, there was no statistically significant difference in any neurological measure (AMS, UEMS, LEMS, ASS-P, or ASS-L) at baseline between the groups (*p* > 0.05; Table 5). AMS, UEMS, ASS-P, and ASS-L, but not LEMS, increased significantly from baseline to 1 year in AIS-A patients in both groups (*p* < 0.01; Table 5). These results suggest that neurologic examinations showed a minor, but significant, increase in both motor and sensory scores over time in complete SCI patients in both groups. However, the mean change of UEMS over 1 year in the transplantation group was significantly greater than that in the control group (7.8 ± 1.1 versus 3.9 ± 0.6; *p* < 0.01) while the mean changes in ASS-P and ASS-L over 1 year were not significantly different in either group (Table 5). No strong correlation between AIS grade conversion and the change in AMS and ASS over 1 year was evident in AIS-A patients in the transplantation group. However, patients 7 and 8, who converted to AIS-C and AIS-B, respectively, showed an increase of 2 points in LEMS and 54 points in ASS-L at 1 year after transplantation, respectively (Tables 7 and 9). In incomplete SCI, two patients in the transplantation group showed greater increase in LEMS and ASS-P at 1 year, compared with those in the control group (Tables 6–9).

### 3.6. Motor Level Recovery

**Figure 4:**
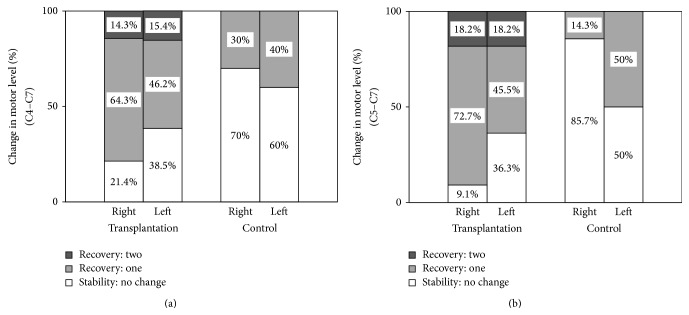
Proportion of AIS-A SCI individuals with initial C4–C7 (a) or C5–C7 (b) motor level remaining stable or gaining motor levels from the baseline to 1-year follow-up in the transplantation and control groups. The cervical motor level is indicated separately in the right and left sides of the cord. The percentage of individuals in each category of motor level change or stability at 1 year after the baseline assessment in both groups is displayed in each bar graph.

Of AIS-A SCI subjects with initial motor levels C4 to C7 in both transplantation and control groups, no patient deteriorated by one or more motor levels over 1 year (Table 4). The proportion of individuals with initial C4–C7 or C5–C7 SCI having a stable or recovering motor level on the right and left side in both groups at 1 year after the baseline assessment is shown in Figure 4. For subjects with initial C5–C7 SCI in the transplantation group, motor level remained the same in 9.1% and 36.3% (right and left side, resp.) and improved by one level in 72.7% and 45.5% and by two levels in 18.2% and 18.2%. AIS grade conversion appeared not to influence motor level changes. However, in the control group, motor level remained the same in 85.7% and 50.0% (right and left side, resp.) and improved by one level in 14.3% and 50.0%, and no patient recovered two levels (Figure 4(b)). The proportion of individuals with initial C4–C7 motor level SCI having a stable or recovering motor level in both groups at 1 year was similar to that of patients with initial C5–C7 motor levels (Figure 4(a)). Thus, a greater proportion of AIS-A patients in the transplantation group recovered one or more motor levels, compared with the control group, at 1 year. There was no zone of partial preservation below the neurological level of injury in complete SCI patients in both groups at baseline.

### 3.7. Electrophysiological Assessments

Complementary to the neurological assessment, electrophysiological measurements provide objective tools for SCI assessment. They provide informative, quantitative data on changes that occur in neural circuitry [47, 48]. A series of SSEP and MEP studies of the upper and lower limbs was conducted for patients in the transplantation group, and detailed data of the latencies and amplitudes of SSEPs and MEPs from patients who showed responses in electrophysiological parameters over 1 year after implantation are summarized in Tables 10 and 11. Following hNSPC transplantation, no patients showed negative changes in neurophysiological measures during follow-up (data not shown). This postoperative longitudinal assessment demonstrates the safety of intraspinal hNSPC injections, even though a relatively large number of cells were injected three times, into the core, rostral and caudal to the lesion.

In the SSEP study of upper limbs, the median and ulnar nerves were stimulated. Of the 17 AIS-A patients, 6 (35.3%) showed positive response at 1 year, while there was no response before transplantation (patients 1, 2, 4, 6, 7, and 10; Table 10). Three patients (patients 5, 8, and 15) showed transient responses in SSEPs during follow-up; however, the cortical response disappeared at 1 year. In the SSEP study of lower limbs, the tibial, peroneal, and pudendal nerves were stimulated. Only one patient (patient 7) who converted to AIS-C showed positive responses in the SSEPs of the tibial and peroneal nerves at 1 year. Another patient (patient 15) who also converted to AIS-C exhibited no response at 1 year although a transient response at 2 months could be observed (Table 10). Thus, in a patient (patient 7) with complete SCI, hNSPC transplantation may repair the injured ascending spinal tract from the upper and lower limbs, which was supported by the SSEP findings, objectively validating conductivity repair in SCI [26]. However, no strong correlation between ASIA motor and sensory scores and SSEP measurements was evident in the transplanted patients. Of the two AIS-B patients, one (patient 19) showed a positive response in the SSEP study of the pudendal nerve at 1 year (Table 10).

Motor evoked responses were measured over the biceps brachii and abductor pollicis brevis muscles for the upper limbs and the tibialis anterior muscle for the lower limbs. Of the 17 AIS-A patients, 10 (58.8%) showed positive response in the MEPs of upper limbs at 1 year, while there was no response before transplantation (patients 1–3, 6, 9, 10, 12, 14, 16, and 17; Table 11). One patient (patient 11) showed a transient response in MEPs in the abductor pollicis brevis muscle during follow-up; however, the response disappeared at 1 year. Unlike the SSEP studies, three patients with AIS grade conversions did not show a positive response in MEPs following transplantation. No strong correlation between ASIA motor and sensory scores and MEP measurements was evident in the transplanted patients. Additionally, in AIS-B patients, the MEP study did not show a positive response at 1 year.

### 3.8. Spinal MRI Findings

**Figure 5:**
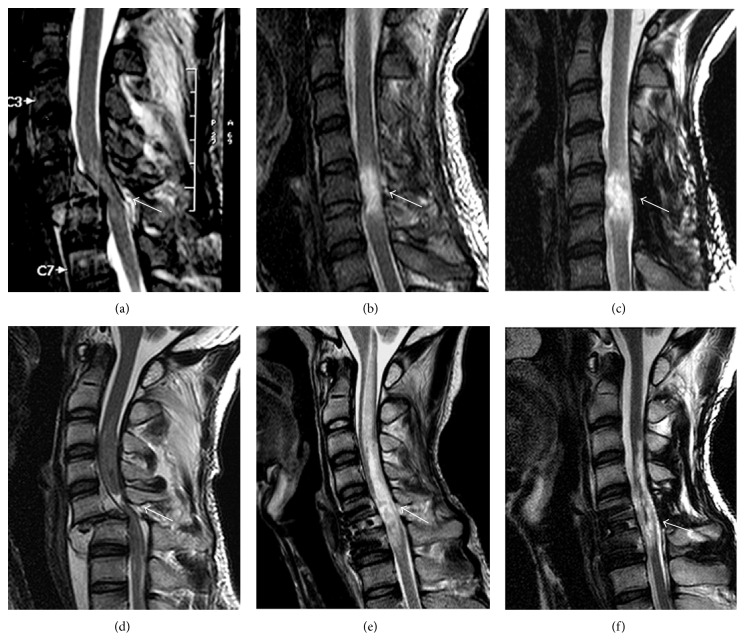
Sagittal T2W MRI scan of two SCI patients (upper panel: patient 8; lower panel: patient 15) at the time of injury (a, d), before transplantation as a baseline (b, e), and 1 year after transplantation (c, f). Follow-up findings showed progressive myelomalacic change at the site of cell transplantation (c) or myelomalacia and atrophy of the cord (f) in cell implantation areas. The white arrows mark the site of injury.

Changes in the MRI findings of patients in the transplantation group are given in Table 12 and Figure 5. Nine of the 19 patients (47.4%) showed progressive posttraumatic myelomalacic change in the spinal cord at the site of cell transplantation; however, three (15.8%) showed a decrease in the diameter of the spinal cord and seven (36.8%) demonstrated no change. The lesion length of SCI was decreased in all patients at 1 year. Other findings, including spinal cord atrophy (3 patients), myelomalacia (17 patients), and cystic degeneration (4 patients), were observed during follow-up. However, no significant change, such as tumor formation or syringomyelia, was found on any of the MRI sequences, at the implantation site, or at any other point in the neuraxis. Additionally, qualitative MRI imaging patterns, the extent of the cord compression, and lesion length in acute SCI appeared not to correlate with neurological or electrophysiological improvements at 1 year after transplantation.

## 4. Discussion

Attempts to induce recovery after SCI by transplanting cells or tissues have been a major focus of much research over the last several decades. Many studies have evaluated the effects of transplanting a wide variety of cell types in SCI animal models and, remarkably, many studies have indicated improved functional outcomes [5]. However, there are difficulties in directly comparing studies because of the varying degree of characterization of the transplanted cells, different injury models, implantation at different time points after SCI, and different evaluation methods. Recent studies have reported that implanted human fetal brain-derived NSPCs can become integrated into injured mice spinal cord and induce locomotor recovery [20, 22, 49]. They have shown that engrafted cells differentiate predominantly into oligodendrocytes and that survival of donor-derived cells is required to sustain locomotor recovery, suggesting that oligodendrocyte integration with the host is likely to be a key mechanism in recovery. This differentiation pattern of donor-derived cells is in contrast to many studies that have demonstrated predominant astroglial fate or differentiation failure following acute or subacute NSPCs transplantation [49].

**Figure 6:**
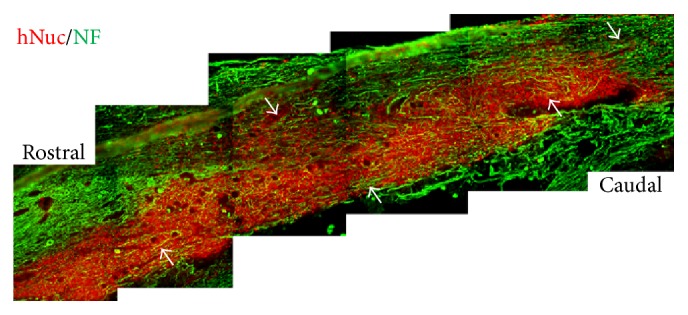
Direct transplantation of hNSPCs into the injured thoracic spinal cord (T9) of adult Sprague-Dawley rats with contusive SCI showed robust long-term engraftment and extensive migration of donor-derived cells and induced host axonal growth along engrafted cells. At 12 weeks after transplantation, immunohistochemistry was conducted in the sectioned spinal cord tissues using anti-human nuclei marker (hNuc) and anti-NF and visualized with fluorescein or Texas red-labeled secondary antibodies. Many hNuc-positive cells (colored red) survived and migrated extensively to rostral and caudal parts of the injury site, including the spared tissue surrounding the lesion. Multiple neuronal processes expressing NF (colored green) extended over the engrafted human cells, as indicated by arrows. Scale bar: 200 *μ*m.

In our preclinical study, we induced contusive thoracic spinal cord injury (T9) in adult Sprague-Dawley rats using the Infinite Horizon Impactor [50] and transplanted hNSPCs used in this study into the epicenter of the injured cord at 7 days following the induction of SCI. Grafted cells exhibited robust engraftment, extensive migration, integration with host cells, and differentiation into neurons and glial cells at 12 weeks after transplant (Figure 6). Additionally, hNSPC-implanted animals showed improved locomotor recovery and no detectable mechanical allodynia (data not shown). On average, 21.3% of donor-derived cells differentiated into neurons, 3.5% into astrocytes, and 1.5% into mature oligodendrocytes. However, more than 80% of engrafted cells expressed the immature cell marker nestin, suggesting that they may still remain as undifferentiated neural precursors. The sum of all quantification markers was more than 100%, suggesting that there is an overlap between some cell markers. Nestin, in particular, has been found to colocalize with *β*-tubulin III, GFAP, and the oligodendroglial progenitor cell marker Olig2 [51]. These findings indicate that in our preclinical study the predominant differentiation of hNSPCs into oligodendroglia and the induction of remyelination may not be a major mechanism of locomotor recovery. Several studies from other labs have also observed limited oligodendroglial differentiation* in vivo* after transplantation [51–53]. Thus, further studies to examine the mechanisms underlying the cell fate determination of transplanted hNSPCs in the injured spinal cord are necessary. In fact, there are many other variables that may be involved in neuronal or glial differentiation of transplanted hNSPCs after SCI, including the source of the human cells, culturing techniques, and cell preparation, as well as potential differences between injury models.

**Figure 7:**
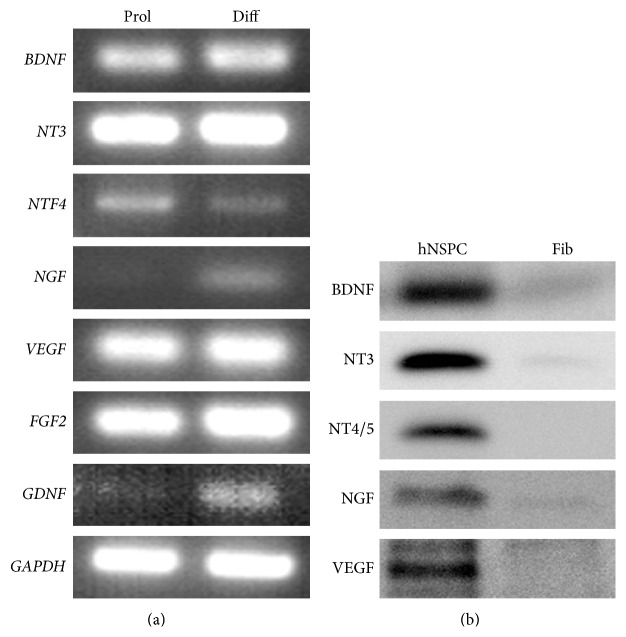
Human NSPCs express diverse trophic factors. (a)* In vitro*, proliferating (Prol) and differentiated (Diff) hNSPCs, respectively, expressed* FGF2*,* GDNF*,* VEGF*, and neurotrophins including* BDNF*,* NT3*,* NTF4*, and* NGF*. (b) hNSPCs notably secreted BDNF, NT3, NT4/5, NGF, and VEGF into the cultured media, compared to human foreskin fibroblasts (Fib).

A comprehensive knowledge of how transplanted hNSPCs exert their therapeutic effects in SCI is still lacking and alternative pathways of hNSPCs-mediated repair should also be considered. Neuronal differentiation of implanted NSPCs could promote restoration of disrupted circuitry by formation of bridges or bypass connections [54] or may provide trophic support, enhancing neuroprotection and regeneration [23]. In a preclinical study, we could also observe that hNSPCs expressed a variety of neurotrophic factors in culture, including BDNF, GDNF, NTF3, NTF4, NGF, VEGF, and FGF2 (Figure 7), and engrafted hNSPCs induced host axonal regrowth in injured spinal cord of rats following transplantation (Figure 6). Thus, neurotrophic factors secreted by implanted hNSPCs may promote host axonal growth along the engrafted cells and contribute to improved locomotor recovery. In addition, recent evidence suggests that transplanted NSPCs can have a variety of effects on the host microenvironment [5]. It is increasingly clear that NSPCs, especially undifferentiated cells, release anti-inflammatory or immune-regulatory molecules at the site of tissue damage, and, in turn, promote functional recovery from CNS injuries [55, 56]. We also observed that a vast majority of hNSPCs remained undifferentiated in injured spinal cord of rats following transplantation which might therefore promote recovery from SCI via a multifaceted response including axonal regeneration, white matter sparing, decrease of glial scar formation or neuronal apoptotic death, or reduction of inflammation.

### 4.1. Safety and Tolerability

In this study, the safety and feasibility of hNSPC transplantation were supported. There was no adverse finding over 1 year after allogeneic hNSPC implantation into patients with cervical SCI. The neurosurgical procedure did not result in any deteriorating sequelae; any ascending damage to one or two spinal cord segments above an injury would be of great clinical significance in cervical injuries. No patient showed serious life-threatening autonomic dysreflexia, worsening of respiratory function, or deterioration in neurological level, sensory or motor function, and urinary bladder complications. Patients also tolerated short-term cyclosporine therapy for immunosuppression. hNSPCs, grown as neurospheres in long-term cultures, did not acquire chromosomal aberrations, maintained their multipotency in neural cell differentiation, and did not introduce pathogens into the cultures. Their transplantation was not obviously associated with any fever, inflammation, immunological rejection, or graft-versus-host reaction. The presence of either undifferentiated NSPCs or inappropriate inflammation may trigger aberrant changes in CNS networks that could lead to neurological dysfunction, such as hyperreflexia, spasticity, dystonia, pain, or allodynia [14, 43]. In this study, hNSPC transplantation tended to increase mean VAS values at 2- and 6-month follow-up times compared to those prior to cell injection, although there was no significant difference between the baseline and follow-up times over 1 year. Additionally, only one patient complained of clinically significant spasticity in both upper and lower extremities; however, he already showed a marked increase in muscle tone of the lower extremities prior to cell implantation. Thus, hNSPC transplantation appeared not to be associated with a greater risk of developing any significant neuropathic pain and spasticity in patients with cervical SCI. Their pain or spasticity was well relieved with medications.

Spine MRIs showed no evidence of any tumor formation, development of posttraumatic syringomyelia, or other adverse radiological findings. However, although this procedure appears safe at 1 year, further follow-up MRIs are necessary to assess the possibility of the development of other abnormalities. MRI has been useful in determining the extent of extrinsic cord compression, outlining qualitative findings, such as cord hemorrhage, edema, soft tissue injury, and hematoma, and in assessing any progressive changes during the course of the trial after SCI. These qualitative MRI imaging parameters have been proposed to correlate with the degree of neurological injury, recovery, and eventual outcome in patients with SCI. However, MRI is still largely a qualitative measure, and quantitative standards, in relation to SCI outcomes, will need to be developed and validated before MRI can be used as an outcome tool [25]. In this study, MRI images were taken with instruments with 1.5 or 3 T field-strength. Higher resolution MRI with a field-strength of 3 T provided better resolution; however, it could not detect transplanted cells. Additionally, we could not find any correlation between qualitative or quantitative MRI findings in acute SCI and neurological outcome at 1 year after transplantation (Table 12). Thus, it is hoped that MRI technologies will develop rapidly such that imaging will become a useful tool for following recovery or predicting outcome of an intervention after SCI.

### 4.2. Neurological Improvements

Spontaneous recovery of neurological function in patients with complete SCI is fairly limited. Prior studies reported spontaneous conversion rates of complete (AIS-A) to incomplete (AIS-B, AIS-C, and AIS-D) status ranging from 4% to 13% [57–61]. The baseline examinations in two of these studies were performed later than 1 week after injury, which would be expected to lower the conversion rate [60, 61]. The recent International Campaign for Cures of Spinal Cord Injury Paralysis (ICCP) systematic review of multiple existing SCI databases reported that about 20% of AIS-A patients on initial acute examination (<1 week after injury) converted to incomplete status during the first postinjury year. However, many patients with SCI show some neurological recovery after the first few days and the AIS grade conversions occurred mostly within the first 2-3 months after injury [62]. It should be noted that a number of variables may also influence the rate of neurological recovery in SCI. These include medical or surgical treatment variables for acute SCI, effect of specialized rehabilitation centers on care for SCI, timing and quality of initial examination of neurological status, formal training and reliability testing of neurologic examiners, and factors affecting the reliability of examinations. For this reason, review of these data should be undertaken with a full understanding of the specific clinical or research context. Additionally, spinal damage in spinal contusions involves not only tract fibers, but also closely packed motor neurons and roots, usually over 2-3 segments that supply arm or leg muscles. Thus, a large part of the motor deficit has to be attributed to the peripheral nervous system, and recovery after complete SCI occurs due to recovery of nerve roots adjacent to the level of the lesion or peripheral axonal sprouting, as well as regeneration of the spinal cord at the level of the lesion [63–65]. However, the influence of extensive damage to peripheral nerves or nerve roots associated with spinal cord contusion on spontaneous recovery is not addressed in the current translational studies or existing SCI databases.

In this study, hNSPC transplantation was not done at the acute stage of SCI, but at an average of 63.4 days after injury, and peripheral nerve or nerve root injuries did not accompany the SCI, as verified by NCS before transplantation. As a result, 18.8% (3/16) of AIS-A patients in the subacute treatment group and 30% (3/10) in the case of the early subacute treatment group improved to AIS-B or AIS-C at 1 year. All three AIS-A patients exhibiting AIS grade conversion were transplanted with hNSPCs in the early subacute stage of SCI. In contrast, only one patient in the control group showed AIS grade conversion. Thus, although further long-term, larger-scale, and randomized clinical trials are required to establish evidence of safety and efficacy, our results suggest that hNSPC transplantation into AIS-A patients within less than 2 months after SCI would result in better neurological outcome. In patients initially assessed as AIS-B, the extent of spontaneous recovery was significantly greater than those in AIS-A; AIS-B conversion to AIS-D has been reported to be as high as 30–40% at 1 year after injury [62]. In this study, both AIS-B patients improved to AIS-D at 1 year following transplantation, albeit the small number of patients. Thus, future clinical trials are required to assess the efficacy of hNSPC transplantation in AIS-B patients to investigate whether AIS-B patients may be a preferable target population to AIS-A patients.

Neurological recovery can also be measured by changes in AMS and ASS, which are often used in phase I/II SCI trials to determine whether an experimental intervention has potentially beneficial effects. Spontaneous motor score recovery in subjects with tetraplegia is also fairly limited. Cervical-injured AIS-A patients showed a mean improvement in AMS of ~8–10 points at 1 year after SCI [62]. However, if changes in AMS are calculated not immediately after SCI, but at different time points after SCI, AIS-A patients are expected to spontaneously improve by 5.7 AMS points, on average, from 8 weeks to the first year after SCI [62]. In this study, we observed a mean improvement of 7.9 AMS points at 1 year after transplantation in AIS-A patients who underwent hNSPC implantation at an average of 63.4 days after injury. The continued improvement of AMS that we observed over 1 year after transplantation is also encouraging (Figure 3). If some recovery was caused by regeneration of injured fibers, this would be expected to be a slow process, similar to what we observed. In contrast, AIS-A patients in the control group showed significantly smaller improvement in AMS compared with the transplantation group (Table 5). Analogous to the findings with motor scores, patients in the cohort showed improvement in sensory scores following transplantation (Figure 3). AIS-B patients also exhibited greater improvement in motor and sensory scores following transplantation, as compared to the control group.

It seems reasonable to suggest that an experimental therapy applied locally to the site of SCI, such as cell transplantation, will have maximum effect within the spinal levels just below the injury. Thus, for patients with complete cervical SCI, an improvement in motor level may readily show a subtle therapeutic effect and could accurately identify a clinically important difference (e.g., improvement in hand function). A number of studies have looked at spontaneous changes in motor levels in patients with SCI. In several studies, the majority of individuals (~55–85%) with complete tetraplegia recovered at least one motor level in the injured cervical cord within 1 year after SCI, whereas, based on initial assessment within 30 days of SCI, only 27% of subjects showed one motor level recovery [34, 60, 66, 67]. This variation in spontaneous recovery rates may be due to different definitions of motor level recovery, different timing of the initial examination, or a different distribution of motor function in the segment below the initial motor level. In this study, the initial examination of motor level was not done at the acute stage of SCI and peripheral nerve injuries did not accompany the SCI. Under this condition, we could observe that higher percentage of AIS-A patients recovered at least one motor level at 1 year after transplantation compared with control group. These results suggest that hNSPC transplantation may improve motor function in spinal segments adjacent to cell injection sites and facilitate endogenous neural substrate repair.

### 4.3. Electrophysiological Evidence of Recovery

Electrophysiological examinations could help in understanding the mechanism(s) of new therapeutic interventions directed at enhancing recovery of spinal cord function. Repair mechanisms, such as remyelination/regeneration and reconnection of damaged spinal tract fibers, may be reflected in changes in spinal impulse conductivity. In a multicenter study, SSEP and MEP recordings remained absent over 1 year in AIS-A patients, and SSEP and MEP latencies remained unchanged over time in AIS-B patients, indicating that neurological and functional recovery in SCI patients were apparently not related to improvements in spinal conductivity [26]. Thus, it was assumed that spontaneous functional recovery occurs primarily through compensation in complete SCI and through neural plasticity in incomplete SCI, rather than through repair of damaged spinal pathways. In this study, electrophysiological recordings showed no response below the level of injury before hNSPC transplantation, confirming the completeness of the SCI. However, we demonstrated a positive response in SSEP and MEP activities in 35.3% and 58.8% of AIS-A patients, respectively, below the level of injury at 1 year after transplantation. Additionally, a patient with complete SCI showed a positive response in SSEP studies of upper and lower limbs following transplantation. In incomplete SCI, a patient also exhibited a positive response in the SSEP study of lower limbs. These findings suggest that hNSPC transplantation may mediate repair across the injury site in the spinal cord. However, the relationship between changes in electrophysiological measures and different outcome tools of recovery has not been fully explored yet, and the hNSPC-mediated recovery mechanisms in complete and incomplete SCI are also still in need of further investigation.

## 5. Conclusion 

Our studies offer support for the safety and tolerability of hNSPC transplantation in sensorimotor or motor complete cervical SCI. At 1 year after cell transplantation, there was no evidence of cord damage, syrinx or tumor formation, neurological deterioration, and exacerbating neuropathic pain or spasticity. There are some indications of efficacy, based on neurological and electrophysiological testing with a limited number of patients that justify moving forward to a larger, controlled clinical trial. Therefore, the transplantation of hNSPCs into cervical SCI is safe and well-tolerated and is of modest neurological benefit up to 1 year after transplants. However, further basic and clinical research is also required to achieve neurological improvement more significantly, identify the maximum safely tolerated or optimal dose of intraspinal grafting of hNSPCs, develop appropriate microsurgical transplantation techniques, evaluate the optimal timing of hNSPC transplantation in SCI, and monitor the long-term safety issue related to hNSPC transplantation.

## Figures and Tables

**Table 1:** Demographic, clinical, and neurological features of the patients.

Transplantation group						Control group					
Patient	Sex	Age (years)	Time (days)^a^	SCI level	AIS grade^b^	Patient	Sex	Age (years)	Time (days)^c^	SCI level	AIS grade^d^
1	M	34	38	C3	A	1	M	37	168	C6	A
2	M	36	46	C3	A	2	M	56	19	C4	A
3	M	33	81	C7	A	3	M	28	7	C6	A
4	M	45	52	C4	A	4	M	29	58	C6	A
5	M	26	141	C4	A	5	M	34	33	C5	A
6	M	56	75	C4	A	6	F	54	92	C5	A
7	F	45	21	C3	A	7	M	41	35	C3	A
8	M	24	48	C5	A	8	F	35	37	C3	A
9	M	53	123	C3	A	9	F	22	60	C3	A
10	M	32	28	C3	A	10	M	28	92	C6	A
11	M	27	59	C5	A	11	M	35	17	C7	A
12	M	54	213	C3	A	12	M	51	97	C3	A
13	M	57	16	C4	A	13	M	24	66	C4	A
14	M	29	34	C4	A	14	M	39	19	C5	B
15	M	23	18	C4	A	15	M	46	38	C7	B
16	F	23	141	C3	A						
17	M	51	24	C4	A						
18	F	41	25	C7	B						
19	M	18	22	C8	B						

Patients are listed according to AIS grade. M = male. F = female. ^a^Time between the injury onset and hNSPC transplantation, ^b^AIS grade before transplantation, ^c^time between the injury onset and the initial evaluation of AIS examination in the hospital, and ^d^AIS grade at the initial evaluation.

**Table 2:** Changes of spasticity in upper extremities of patients with cervical SCI using modified Ashworth scale.

Patient	Before transplantation		2 months^a^		6 months^b^		1 year^c^	
	Right	Left	Right	Left	Right	Left	Right	Left
1	0	0	1	1	1	1	1+	1+
2	0	0	0	0	1	1	0	0
3	0	0	0	0	0	0	0	0
4	0	0	0	0	0	0	1	0
5	1	1	0	0	1	1	1	1
6	0	0	1	1	1	0	1	1
7	0	0	0	0	0	0	0	0
8	0	0	0	0	1	1	0	0
9	0	0	1+	1+	1	1	1	1
10	0	0	1	1	2	2	1+	1+
11	0	0	0	0	0	0	0	0
12	0	0	0	0	1	1	1	1
13	0	0	0	0	0	0	0	0
14	0	0	0	0	1+	1+	2	2
15	0	0	0	0	0	0	0	0
16	0	0	1	1	1	1	1+	1+
17	0	0	0	0	1	1	2	1
18	0	0	0	0	0	0	0	0
19	0	0	0	0	0	0	0	0

^a,b,c^2, 6, and 12 months after hNSPC transplantation.

**Table 3:** Changes of spasticity in lower extremities of patients with cervical SCI using modified Ashworth scale.

Patient	Before transplantation		2 months^a^		6 months^b^		1 year^c^	
	Right	Left	Right	Left	Right	Left	Right	Left
1	1+	1+	1+	1+	1+	1+	1+	1+
2	0	0	0	0	1	1	1	1
3	1	1	1	1	1	1	1	1
4	1	1	1	1	1+	1+	1+	1+
5	1	1	1	1	1	1	1	1
6	0	0	1	1	0	0	1	1
7	0	0	1	1	1	1	1	1
8	1	1	1	1	2	2	1	1
9	1	1	1+	1+	1+	1+	1	1
10	0	0	0	0	2	1+	2	1+
11	0	0	0	0	1	1	1	1
12	0	0	0	0	1	1	1	1
13	0	0	0	0	1	1	1	1
14	2	2	1+	1+	2	2	2	2
15	1+	1+	1	1	1+	1+	2	2
16	1	1	1	1	1	1	1	1
17	1	1	1	1	1	1	2	2
18	1	1	1+	1+	1+	1+	1+	1+
19	0	0	1+	1+	1+	1+	2	2

^a,b,c^2, 6, and 12 months after hNSPCs transplantation.

**Table 4:** AIS grade conversion and motor level changes after cervical SCI.

Transplantation group					Control group				
Patient	Before AIS^a^	After AIS^b^	Prelevel (R/L)^c^	Postlevel (R/L)^d^	Patient	Initial AIS^e^	1 year AIS^f^	Initial level (R/L)^g^	1 year level (R/L)^h^
1	A	A	C4/—^i^	C4/C4	1	A	A	C7/C6	C7/C7
2	A	A	—^i^/—^i^	—/—	2	A	A	C4/C4	C4/C4
3	A	A	C7/C7	C8/C8	3	A	A	C6/C6	C7/C7
4	A	A	C5/C5	C6/C6	4	A	A	C6/C6	C6/C6
5	A	A	C5/C6	C6/C6	5	A	A	C6/C5	C6/C6
6	A	A	C4/C4	C4/C4	6	A	B	C6/C6	C6/C6
7	A	C	C5/C5	C7/C6	7	A	A	—/—	—/—
8	A	B	C5/C6	C6/C6	8	A	A	C4/C4	C5/C4
9	A	A	C5/C5	C5/C5	9	A	A	—/—	C4/C4
10	A	A	—/—	C5/—	10	A	A	C6/C6	C6/C6
11	A	A	C6/C7	C7/C8	11	A	A	C7/C7	C7/C7
12	A	A	C4/—	C5/—	12	A	A	—/—	—/—
13	A	A	C5/C5	C7/C7	13	A	A	C4/C4	C5/C5
14	A	A	C5/C5	C6/C6	14	B	B	C5/C6	C6/C7
15	A	C	C5/C5	C6/C5	15	B	B	C7/C7	C8/C8
16	A	A	—/C4	C5/C5					
17	A	A	C5/C4	C6/C6					
18	B	D	C5/C5	C8/C8					
19	B	D	C8/C8	T1/T1					

^a^AIS grade before transplantation, ^b^AIS grade 1 year after transplantation, ^c^motor level before transplantation (R/L: right side/left side), ^d^motor level 1 year after transplantation, ^e^AIS grade at the initial evaluation of AIS examination in the hospital after SCI, ^f^AIS grade 1 year after initial assessment, ^g^motor level at the initial evaluation, ^h^motor level 1 year after initial evaluation, and ^i^—motor levels C1 to C3. There was no zone of partial preservation below the neurological level of injury in complete SCI patients in both groups at baseline.

**Table 5 tab5:** Summary of AMS and ASS changes in patients with complete cervical SCI.

	Transplantation group			Control group			*p* ^j^
	Prescore^a,g^	Postscore^b,h^	After-before^c^	Initial score^d^	1 year score^e^	Initial 1 year^f,i^	
AMS	9.5 ± 2.1^k^	17.4 ± 2.7	7.9 ± 1.2	15.5 ± 2.1	19.5 ± 2.1	3.9 ± 0.6	0.013
UEMS	9.5 ± 2.1	17.2 ± 2.7	7.8 ± 1.1	15.5 ± 2.1	19.5 ± 2.1	3.9 ± 0.6	0.014
LEMS	0.0 ± 0.0	0.1 ± 0.1	0.1 ± 0.1	0.0 ± 0.0	0.0 ± 0.0	0.0 ± 0.0	0.391
ASS-P	14.1 ± 1.1	18.9 ± 1.6	4.8 ± 1.3	19.4 ± 2.8	22.3 ± 2.9	2.9 ± 0.6	0.551
ASS-L	14.7 ± 1.2	21.6 ± 3.7	6.9 ± 3.1	19.5 ± 2.8	21.8 ± 2.9	2.3 ± 0.5	0.309

^a^AMS/ASS before transplantation, ^b^AMS/ASS 1 year after transplantation, ^c^AMS/ASS changes over 1-year follow-up period, ^d^AMS/ASS at the initial evaluation in the hospital after SCI, ^e^AMS/ASS 1 year after initial evaluation, ^f^AMS/ASS changes over 1-year follow-up period, ^g^ *p *> 0.05, between pre-AMS/ASS in the transplantation group and initial AMS/ASS in the control group, ^h^ *p *< 0.01, between before AMS/ASS and after AMS/ASS in the transplantation group, ^i^ *p *< 0.01, between initial AMS/ASS and 1-year after AMS/ASS in the control group, ^j^ *p*-values, between AMS/ASS changes in both transplantation and control groups, and ^k^mean ± SEM.

**Table 6 tab6:** AMS upper extremity motor score (UEMS).

Transplantation group						Control group			
Patient	Before^a^	2 months^b^	6 months^c^	1 year^d^	UEMS changes^e^	Patient	Initial^f^	1 year^g^	UEMS changes^h^
1	0	0	0	6	6	1	25	27	2
2	0	3	5	8	8	2	14	15	1
3	29	37	38	38	9	3	21	26	5
4	12	23	23	24	12	4	21	26	5
5	13	15	18	18	5	5	17	21	4
6	2	2	3	3	1	6	20	23	3
7	15	18	22	27	12	7	5	13	8
8	18	18	20	24	6	8	11	14	3
9	6	6	6	6	0	9	9	11	2
10	0	2	4	5	5	10	23	24	1
11	26	33	36	36	10	11	26	33	7
12	1	1	1	7	6	12	2	7	5
13	10	20	26	32	22	13	8	13	5
14	9	17	18	18	9	14	14	25	11
15	9	19	16	15	6	15	30	38	8
16	3	6	6	10	7				
17	8	10	12	16	8				
18	28	42	44	44	16				
19	44	50	50	50	6				

^a^UEMS before transplantation, ^b,c,d^ UEMS 2, 6, and 12 months after transplantation, ^e^UEMS change over 1-year follow-up period, ^f^UEMS at the initial evaluation in the hospital after SCI, ^g^UEMS 1 year postinitial evaluation, and ^h^UEMS change over 1-year follow-up period.

**Table 7 tab7:** AMS lower extremity motor score (LEMS).

Transplantation group						Control group			
Patient	Before	2 months	6 months	1 year	LEMS changes	Patient	Initial	1 year	LEMS changes
1	0	0	0	0	0	1	0	0	0
2	0	0	0	0	0	2	0	0	0
3	0	0	0	0	0	3	0	0	0
4	0	0	0	0	0	4	0	0	0
5	0	0	0	0	0	5	0	0	0
6	0	0	0	0	0	6	0	0	0
7	0	0	0	2	2	7	0	0	0
8	0	0	0	0	0	8	0	0	0
9	0	0	0	0	0	9	0	0	0
10	0	0	0	0	0	10	0	0	0
11	0	0	0	0	0	11	0	0	0
12	0	0	0	0	0	12	0	0	0
13	0	0	0	0	0	13	0	0	0
14	0	0	0	0	0	14	0	0	0
15	0	0	0	0	0	15	0	0	0
16	0	0	0	0	0				
17	0	0	0	0	0				
18	0	21	32	32	32				
19	0	17	22	22	22				

**Table 8 tab8:** ASS-pin prick (ASS-P).

Transplantation group						Control group			
Patient	Before	2 months	6 months	1 year	ASS-P changes	Patient	Initial	1 year	ASS-P changes
1	12	12	12	12	0	1	32	33	1
2	11	11	11	11	0	2	13	14	1
3	25	25	25	25	0	3	29	33	4
4	13	13	13	14	1	4	33	35	2
5	16	18	18	18	2	5	16	16	0
6	12	14	14	14	2	6	28	33	5
7	13	17	17	22	9	7	8	10	2
8	19	19	20	24	5	8	11	18	7
9	9	12	12	12	3	9	9	16	7
10	8	9	9	14	6	10	28	30	2
11	22	22	24	24	2	11	30	34	4
12	10	10	12	12	2	12	8	9	1
13	16	16	18	18	2	13	7	9	2
14	16	18	24	37	21	14	45	56	11
15	12	26	18	22	10	15	56	60	4
16	12	12	14	16	4				
17	14	16	18	26	12				
18	31	40	46	46	15				
19	38	38	43	70	32				

**Table 9:** ASS-light touch (ASS-L).

Transplantation group						Control group			
Patient	Before	2 months	6 months	1 year	ASS-L changes	Patient	Initial	1 year	ASS-L changes
1	10	12	12	12	2	1	32	33	1
2	11	12	13	13	2	2	13	13	0
3	25	26	26	26	1	3	28	32	4
4	13	19	19	19	6	4	33	37	4
5	17	17	18	18	1	5	16	16	0
6	12	14	14	14	2	6	30	31	1
7	13	17	18	19	6	7	8	12	4
8	22	22	48	76	54	8	11	14	3
9	12	12	12	12	0	9	10	14	4
10	8	10	14	15	7	10	30	32	2
11	21	23	24	26	5	11	28	32	4
12	11	12	12	12	1	12	8	8	0
13	19	19	19	19	0	13	6	9	3
14	19	19	29	40	21	14	46	54	8
15	10	15	16	16	6	15	54	61	7
16	11	12	15	15	4				
17	16	16	16	16	0				
18	68	68	70	70	2				
19	65	90	92	93	28				

**Table 10:** Latency measured by evoked potentials of the upper and lower limbs in patients with cervical SCI who showed response of SSEP over 1 year after hNSPC transplantation.

Patient	Before		2 months		6 months		1 year	
	Right^a^	Left^b^	Right	Left	Right	Left	Right	Left
	Median nerve							
1	0.00^c^	0.00	0.00	0.00	0.00	0.00	19.00	17.95
2	0.00	0.00	0.00	0.00	33.15	22.95	25.15	31.50
10	0.00	0.00	17.80	18.75	19.40	16.00	17.71	20.95
15^d^	0.00	0.00	19.00	18.65	0.00	0.00	0.00	0.00

	Ulnar nerve							
1	0.00	0.00	0.00	0.00	0.00	0.00	23.40	28.95
2	0.00	0.00	0.00	0.00	26.40	0.00	24.85	0.00
4	0.00	0.00	0.00	0.00	37.30	35.30	22.20	23.95
6	0.00	0.00	16.10	0.00	18.05	0.00	18.50	0.00
7	0.00	0.00	0.00	0.00	0.00	0.00	19.35	20.65
10	0.00	0.00	20.10	25.50	18.85	19.35	17.05	20.25
5^d^	0.00	0.00	0.00	0.00	20.90	21.60	0.00	0.00
8^d^	0.00	0.00	27.95	27.95	0.00	0.00	0.00	0.00
15^d^	0.00	0.00	19.45	18.15	0.00	0.00	0.00	0.00

	Tibial nerve							
7	0.00	0.00	0.00	0.00	0.00	0.00	61.90	43.10
15^d^	0.00	0.00	42.70	44.70	0.00	0.00	0.00	0.00

	Peroneal nerve							
7	0.00	0.00	0.00	0.00	0.00	0.00	44.80	44.60
15^d^	0.00	0.00	39.10	48.00	0.00	0.00	0.00	0.00

	Pudendal nerve^e^							
7	0.00		0.00		0.00		61.80	
19	0.00		0.00		44.40		48.00	

^a^Right side, ^b^left side, ^c^ms, and ^d^patients showed recovery of SSEPs at 2- or 6-month follow-up times. However, the cortical response to peripheral stimulation disappeared at 1 year after transplantation. ^e^There is no division into right and left side in pudendal nerve stimulation.

**Table 11:** Latency and amplitude measured by evoked potentials of the upper limbs in patients with cervical SCI who showed response of MEP over 1 year after hNSPC transplantation.

Patient	Before		2 months		6 months		1 year	
	Right^a^	Left^b^	Right	Left	Right	Left	Right	Left
	Biceps brachii muscle (latency)							
6	0.00^c^	0.00	0.00	0.00	14.49	14.31	15.99	17.37
9	0.00	0.00	13.01	13.27	14.57	14.40	14.50	14.60
10	0.00	0.00	0.00	0.00	12.39	0.00	12.16	0.00

	Biceps brachii muscle (amplitude)							
1	0.00^d^	0.00	0.00	0.00	0.79	0.87	0.69	0.61
2	0.00	0.00	0.00	0.00	0.22	0.00	0.62	0.00
6	0.00	0.00	0.00	0.00	0.75	1.25	0.88	0.53
9	0.00	0.00	0.36	0.20	0.65	0.40	0.84	0.54
10	0.00	0.00	0.00	0.00	1.37	0.00	1.66	0.00
12	0.00	0.00	0.00	0.00	0.00	0.00	0.51	0.42
14	0.55	0.00	0.62	0.38	2.14	1.39	1.07	0.66
16	0.00	0.00	0.85	0.76	0.91	2.49	1.57	1.00
17	0.00	0.00	0.82	0.84	0.93	1.24	1.21	1.58

	Abductor pollicis brevis muscle (latency)							
3	0.00	0.00	24.85	0.00	25.95	0.00	28.59	0.00
11^e^	0.00	0.00	0.00	0.00	0.00	30.06	0.00	0.00

	Abductor pollicis brevis muscle (amplitude)							
3	0.00	0.00	0.39	0.00	0.07	0.00	0.18	0.00
11^e^	0.00	0.00	0.00	1.06	0.00	0.00	0.00	0.00

^a^Right side, ^b^left side, ^c^ms, ^d^microvolts, and ^e^the patient showed recovery of MEPs at 2- or 6-month follow-up times. However, the peripheral response to transcranial stimulation disappeared at 1 year after transplantation.

**Table 12:** Changes in spinal MRI findings in patients with cervical SCI before and 1 year after hNSPC transplantation.

Patient	Before transplantation			1 year after transplantation		
	Types^a^	Location	Lesion length	Types^b^	Location	Lesion length
1	I	C2.3–C7.1	81.2 mm	B, C	C4.1–C5.2	29.3 mm
2	I	C2.3–C4.2	33.5 mm	A, B	C3.2–C4.1	12.3 mm
3	III	C5.1–C7.1	30.5 mm	B	C6.2–C6.3	10.3 mm
4	IV	C3.1–C7.2	70.3 mm	B	C5.2–C7.2	32.9 mm
5	IV	C2.1–C7.2	99.6 mm	B	C5.1–C6.3	37.5 mm
6	N/A^c^	C3.3–C5.3	34.6 mm	B	C3.3–C5.2	33.8 mm
7	II	C3.3–C7.1	62.2 mm	B	C5.1–C6.1	24.2 mm
8	IV	C2.1–C7.3	57.1 mm	B	C5.1–C6.1	25.4 mm
9	N/A^c^	C3.3–C4.1	13.8 mm	B	C3.3–C4.1	12.6 mm
10	III	C2.3–C5.3	59.9 mm	C	C3.2–C4.1	13.5 mm
11	IV	C4.3–T1.3	76.7 mm	B, C	C6.1–T1.2	42.9 mm
12	I	C2.1–C5.3	70.0 mm	B	C2.3–C4.3	43.0 mm
13	IV	C3.1–T2.1	101.6 mm	B	C5.2–C6.2	19.3 mm
14	III	C4.2–T1.1	58.4 mm	B	C5.2–C7.1	36.0 mm
15	IV	C2.3–C7.1	89.8 mm	A, B	C3.2–C6.2	59.4 mm
16	I	C2.3–C6.2	67.6 mm	A, B	C4.1–C6.1	41.8 mm
17	III	C4.2–T1.1	84.0 mm	B	C5.1–C6.3	33.7 mm
18	III	C5.2–T1.1	42.3 mm	C	C7.1–C7.2	9.0 mm
19	III	C6.1–T1.2	40.7 mm	B	C7.2	6.9 mm

^a^Types of acute SCI pattern: Type I: hemorrhage, Type II: edema, Type III: mixed or contusion, and Type IV: compression. ^b^Types of subacute and chronic SCI pattern: type A: atrophy, type B: myelomalacia, type C: cyst, and type D; syrinx. ^c^Spine MRI studies were not available in the acute phase of SCI; however, MRI scan showed myelomalacia before hNSPC transplantation.

## References

[1] Baptiste D. C., Fehlings M. G. (2007). Update on the treatment of spinal cord injury. *Progress in Brain Research*.

[2] Barnabé-Heider F., Frisén J. (2008). Stem cells for spinal cord repair. *Cell Stem Cell*.

[3] Sahni V., Kessler J. A. (2010). Stem cell therapies for spinal cord injury. *Nature Reviews Neurology*.

[4] Féron F., Perry C., Cochrane J. (2005). Autologous olfactory ensheathing cell transplantation in human spinal cord injury. *Brain*.

[5] Hernández J., Torres-Espín A., Navarro X. (2011). Adult stem cell transplants for spinal cord injury repair: current state in preclinical research. *Current Stem Cell Research & Therapy*.

[6] Knoller N., Auerbach G., Fulga V. (2005). Clinical experience using incubated autologous macrophages as a treatment for complete spinal cord injury: phase I study results. *Journal of Neurosurgery: Spine*.

[7] Lima C., Escada P., Pratas-Vital J. (2010). Olfactory mucosal autografts and rehabilitation for chronic traumatic spinal cord injury. *Neurorehabilitation and neural repair*.

[8] Mackay-Sim A., Féron F., Cochrane J. (2008). Autologous olfactory ensheathing cell transplantation in human paraplegia: a 3-year clinical trial. *Brain*.

[9] Moviglia G. A., Fernandez Viña R., Brizuela J. A. (2006). Combined protocol of cell therapy for chronic spinal cord injury. Report on the electrical and functional recovery of two patients. *Cytotherapy*.

[10] Saberi H., Moshayedi P., Aghayan H.-R. (2008). Treatment of chronic thoracic spinal cord injury patients with autologous Schwann cell transplantation: an interim report on safety considerations and possible outcomes. *Neuroscience Letters*.

[11] Syková E., Homola A., Mazanec R. (2006). Autologous bone marrow transplantation in patients with subacute and chronic spinal cord injury. *Cell Transplantation*.

[12] Seung H. Y., Yu S. S., Yong H. P. (2007). Complete spinal cord injury treatment using autologous bone marrow cell transplantation and bone marrow stimulation with granulocyte macrophage-colony stimulating factor: phase I/II clinical trial. *Stem Cells*.

[13] Lindvall O., Kokaia Z. (2010). Stem cells in human neurodegenerative disorders—time for clinical translation?. *Journal of Clinical Investigation*.

[14] Hofstetter C. P., Holmström N. A. V., Lilja J. A. (2005). Allodynia limits the usefulness of intraspinal neural stem cell grafts; directed differentiation improves outcome. *Nature Neuroscience*.

[15] Karimi-Abdolrezaee S., Eftekharpour E., Wang J., Morshead C. M., Fehlings M. G. (2006). Delayed transplantation of adult neural precursor cells promotes remyelination and functional neurological recovery after spinal cord injury. *The Journal of Neuroscience*.

[16] Keirstead H. S., Nistor G., Bernal G. (2005). Human embryonic stem cell-derived oligodendrocyte progenitor cell transplants remyelinate and restore locomotion after spinal cord injury. *The Journal of Neuroscience*.

[17] Lu P., Jones L. L., Snyder E. Y., Tuszynski M. H. (2003). Neural stem cells constitutively secrete neurotrophic factors and promote extensive host axonal growth after spinal cord injury. *Experimental Neurology*.

[18] Teng Y. D., Lavik E. B., Qu X. (2002). Functional recovery following traumatic spinal cord injury mediated by a unique polymer scaffold seeded with neural stem cells. *Proceedings of the National Academy of Science of the United States of America*.

[19] Ziv Y., Avidan H., Pluchino S., Martino G., Schwartz M. (2006). Synergy between immune cells and adult neural stem/progenitor cells promotes functional recovery from spinal cord injury. *Proceedings of the National Academy of Sciences of the United States of America*.

[20] Cummings B. J., Uchida N., Tamaki S. J. (2005). Human neural stem cells differentiate and promote locomotor recovery in spinal cord-injured mice. *Proceedings of the National Academy of Sciences of the United States of America*.

[21] Salazar D. L., Uchida N., Hamers F. P. T., Cummings B. J., Anderson A. J. (2010). Human neural stem cells differentiate and promote locomotor recovery in an early chronic spinal cord injury NOD-scid mouse model. *PLoS ONE*.

[22] Hooshmand M. J., Sontag C. J., Uchida N., Tamaki S., Anderson A. J., Cummings B. J. (2009). Analysis of host-mediated repair mechanisms after human CNS-stem cell transplantation for spinal cord injury: correlation of engraftment with recovery. *PLoS ONE*.

[23] Yan J., Xu L., Welsh A. M. (2007). Extensive neuronal differentiation of human neural stem cell grafts in adult rat spinal cord. *PLoS Medicine*.

[24] Globe Newswire http://investor.stemcellsinc.com/phoenix.zhtml?c=86230&p=irol-newsArticle&ID=1974747.

[25] Steeves J. D., Lammertse D., Curt A. (2007). Guidelines for the conduct of clinical trials for spinal cord injury (SCI) as developed by the ICCP panel: clinical trial outcome measures. *Spinal Cord*.

[26] Curt A., Van Hedel H. J. A., Klaus D., Dietz V. (2008). Recovery from a spinal cord injury: significance of compensation, neural plasticity, and repair. *Journal of Neurotrauma*.

[27] Ditunno J. F., Flanders A. E., Kirshblum S., Kirshblum S., Campagnolo D. I., Delisa J. A. (2002). Predicting outcome in traumatic spinal cord injury. *Spinal Cord Medicine*.

[28] Dumitru D., Amato A. A., Zwarts M. J. (2002). *Electrodiagnostic Medicine*.

[29] Kim H.-T., Kim I.-S., Lee I.-S., Lee J.-P., Snyder E. Y., In Park K. (2006). Human neurospheres derived from the fetal central nervous system are regionally and temporally specified but are not committed. *Experimental Neurology*.

[30] Shinawi M., Cheung S. W. (2008). The array CGH and its clinical applications. *Drug Discovery Today*.

[31] Lee H., Yun S., Kim I.-S. (2014). Human fetal brain-derived neural stem/progenitor cells grafted into the adult epileptic brain restrain seizures in rat models of temporal lobe epilepsy. *PLoS ONE*.

[32] Kokaia Z., Martino G., Schwartz M., Lindvall O. (2012). Cross-talk between neural stem cells and immune cells: the key to better brain repair?. *Nature Neuroscience*.

[33] Titomanlio L., Kavelaars A., Dalous J. (2011). Stem cell therapy for neonatal brain injury: perspectives and Challenges. *Annals of Neurology*.

[34] Steeves J. D., Kramer J. K., Fawcett J. W. (2011). Extent of spontaneous motor recovery after traumatic cervical sensorimotor complete spinal cord injury. *Spinal Cord*.

[35] Ramón S., Domínguez R., Ramírez L. (1997). Clinical and magnetic resonance imaging correlation in acute spinal cord injury. *Spinal Cord*.

[36] Potter K., Saifuddin A. (2003). Pictorial review: MRI of chronic spinal cord injury. *British Journal of Radiology*.

[37] Flanders A. E., Spettell C. M., Tartaglino L. M., Friedman D. P., Herbison G. J. (1996). Forecasting motor recovery after cervical spinal cord injury: value of MR imaging. *Radiology*.

[38] Jensen M. P., Karoly P., Turk D. C., Melzack R. (1992). Self-report scales and procedures for assessing pain in adults. *Handbook of Pain Assessment*.

[39] Pandyan A. D., Johnson G. R., Price C. I. M., Curless R. H., Barnes M. P., Rodgers H. (1999). A review of the properties and limitations of the Ashworth and modified Ashworth Scales as measures of spasticity. *Clinical Rehabilitation*.

[40] Spandidos A., Wang X., Wang H., Seed B. (2009). PrimerBank: a resource of human and mouse PCR primer pairs for gene expression detection and quantification. *Nucleic Acids Research*.

[41] Barrett H., McClelland J. M., Rutkowski S. B., Siddall P. J. (2003). Pain characteristics in patients admitted to hospital with complications after spinal cord injury. *Archives of Physical Medicine and Rehabilitation*.

[42] Noonan V. K., Kopec J. A., Zhang H., Dvorak M. F. (2008). Impact of associated conditions resulting from spinal cord injury on health status and quality of life in people with traumatic central cord syndrome. *Archives of Physical Medicine and Rehabilitation*.

[43] Macias M. Y., Syring M. B., Pizzi M. A., Crowe M. J., Alexanian A. R., Kurpad S. N. (2006). Pain with no gain: allodynia following neural stem cell transplantation in spinal cord injury. *Experimental Neurology*.

[44] Hitzig S. L., Tonack M., Campbell K. A. (2008). Secondary health complications in an aging canadian spinal cord injury sample. *American Journal of Physical Medicine & Rehabilitation*.

[45] Lechner H. E., Frotzler A., Eser P. (2006). Relationship between self- and clinically rated spasticity in spinal cord injury. *Archives of Physical Medicine and Rehabilitation*.

[46] Marino R. J., Barros T., Biering-Sorensen F. (2003). International standards for neurological classification of spinal cord injury. *The journal of spinal cord medicine.*.

[47] Curt A., Dietz V. (1999). Electrophysiological recordings in patients with spinal cord injury: significance for predicting outcome. *Spinal Cord*.

[48] Xie J., Boakye M. (2008). Electrophysiological outcomes after spinal cord injury. *Neurosurgical Focus*.

[49] Salazar D. L., Uchida N., Hamers F. P. T., Cummings B. J., Anderson A. J. (2010). Human neural stem cells differentiate and promote locomotor recovery in an early chronic spinal cord injury NOD-*scid* mouse model. *PLoS ONE*.

[50] Cheriyan T., Ryan D. J., Weinreb J. H. (2014). Spinal cord injury models: a review. *Spinal Cord*.

[51] Åkesson E., Piao J.-H., Samuelsson E.-B. (2007). Long-term culture and neuronal survival after intraspinal transplantation of human spinal cord-derived neurospheres. *Physiology & Behavior*.

[52] Svendsen C. N., Caldwell M. A., Ostenfeld T. (1999). Human neural stem cells: Isolation, expansion and transplantation. *Brain Pathology*.

[53] Zhang S.-C., Wernig M., Duncan I. D., Brüstle O., Thomson J. A. (2001). In vitro differentiation of transplantable neural precursors from human embryonic stem cells. *Nature Biotechnology*.

[54] Abematsu M., Tsujimura K., Yamano M. (2010). Neurons derived from transplanted neural stem cells restore disrupted neuronal circuitry in a mouse model of spinal cord injury. *The Journal of Clinical Investigation*.

[55] Bacigaluppi M., Pluchino S., Jametti L. P. (2009). Delayed post-ischaemic neuroprotection following systemic neural stem cell transplantation involves multiple mechanisms. *Brain*.

[56] Pluchino S., Quattrini A., Brambilla E. (2003). Injection of adult neurospheres induces recovery in a chronic model of multiple sclerosis. *Nature*.

[57] Burns A. S., Lee B. S., Ditunno J. F., Tessler A. (2003). Patient selection for clinical trials: the reliability of the early spinal cord injury examination. *Journal of Neurotrauma*.

[58] Consortium for Spinal Cord Medicine (2003). Outcomes following traumatic spinal cord injury: clinical practice guidelines for health-care professionals. *Journal of Spinal Cord Medicine*.

[59] Marino R. J., Ditunno J. F., Donovan W. H., Maynard F. (1999). Neurologic recovery after traumatic spinal cord injury: data from the model spinal cord injury systems. *Archives of Physical Medicine and Rehabilitation*.

[60] Waters R. L., Adkins R. H., Yakura J. S., Sie I. (1993). Motor and sensory recovery following complete tetraplegia. *Archives of Physical Medicine and Rehabilitation*.

[61] Waters R. L., Adkins R. H., Yakura J. S., Sie I. (1992). Recovery following complete paraplegia. *Archives of Physical Medicine and Rehabilitation*.

[62] Fawcett J. W., Curt A., Steeves J. D. (2007). Guidelines for the conduct of clinical trials for spinal cord injury as developed by the ICCP panel: spontaneous recovery after spinal cord injury and statistical power needed for therapeutic clinical trials. *Spinal Cord*.

[63] Dietz V., Curt A. (2006). Neurological aspects of spinal-cord repair: promises and challenges. *The Lancet Neurology*.

[64] Ditunno J. F., Flanders A. E., Kirshblum S., Graziani V., Tessler A., Kirshblum S., Campagnolo D. I., Delisa J. A. (2002). Predicting outcome in traumatic spinal cord injury. *Spinal Cord Medicine*.

[65] Marino R. J., Herbison G. J., Ditunno J. F. (1994). Peripheral sprouting as a mechanism for recovery in the zone of injury in acute quadriplegia: a single-fiber EMG study. *Muscle and Nerve*.

[66] Ditunno J. F., Cohen M. E., Hauck W. W., Jackson A. B., Sipski M. L. (2000). Recovery of upper-extremity strength in complete and incomplete tetraplegia: a multicenter study. *Archives of Physical Medicine and Rehabilitation*.

[67] Fisher C. G., Noonan V. K., Smith D. E., Wing P. C., Dvorak M. F., Kwon B. (2005). Motor recovery, functional status, and health-related quality of life in patients with complete spinal cord injuries. *Spine*.

